# Ticagrelor or dipyridamole plus aspirin may be a promising antiplatelet therapy in patients with minor stroke or transient ischemic attack: a bayesian network meta-analysis

**DOI:** 10.3389/fphar.2025.1561564

**Published:** 2025-03-26

**Authors:** Shiran Qin, Si Gao, Dandan Xu, Li Zhang, Yanmei Luo, Sitong Guo

**Affiliations:** Department of Pharmacy, Guangxi Academy of Medical Sciences and the People’s Hospital of Guangxi Zhuang Autonomous Region, Nanning, China

**Keywords:** minor stroke, TIA, antiplatelet, network meta-analysis, Bayesian

## Abstract

**Background:**

The efficacy and safety of different antiplatelet in minor strokes or transient ischemic attacks (TIAs) remains controversial.

**Methods:**

We searched PubMed, Embase, Web of Science and the Cochrane Library to identify all eligible articles until 12 September 2024. Efficacy outcomes were all-cause mortality, excellent outcome, functional independence and recurrent stroke. Safety outcomes were any types of bleeding and intracerebral hemorrhage (ICH). The associations were calculated for the overall data by using the odds ratios (ORs).

**Results:**

12 high-quality studies with 10 RCTs and 2 Non-RCTs were included, involving 61,281 patients with minor strokes or TIAs. Ticagrelor + Aspirin was significantly more effective than Clopidogrel + Aspirin in preventing post-stroke neurological dysfunctions (mRS 0–1), recurrent stroke and major vascular events for up to 90 days. But Ticagrelor + Aspirin is associated with an increased risk of any bleeding and mild bleeding at 90 days, and there is no significant difference in other bleeding risks. The risk of any bleeding in Dipyridamole + Aspirin is not significantly different from that in Aspirin, and is even significantly lower than in Ticagrelor. Compared with other dual antiplatelet therapies (DAPTs), Dipyridamole + Aspirin had no significant difference in the risk of all-cause mortality and major vascular events during follow-up.

**Conclusion:**

For minor strokes or TIAs with a low bleeding risk or CYP2C19 loss-of-function alleles, Ticagrelor + Aspirin may be a better choice than Clopidogrel + Aspirin. Due to limited studies, the superiority of Dipyridamole + Aspirin is still difficult to conclude, and further high-quality studies are needed to verify the benefits of Dipyridamole + Aspirin in minor stroke or TIAs.

**Systematic Review Registration:**

https://www.crd.york.ac.uk/prospero/, identifier CRD42024537462

## 1 Introduction

Stroke is an acute cerebrovascular disease, encompassing both ischemic and hemorrhagic types. It is the second leading cause of death and disability among adults globally, characterized by high morbidity, disability, mortality, and recurrence rates (Wang W. et al., 2017; Wu et al., 2019). Acute ischemic stroke (AIS) represents approximately 70%–85% of all strokes, with transient ischemic attack (TIA) and minor stroke comprising about 65% of these cases (Wang et al., 2013). The risk of recurrent stroke and other vascular events is notably high in the early stages, ranging from 3.7% to 11.7% within the first 2 weeks following onset (Wang et al., 2013; Johnston et al., 2018; Amarenco et al., 2016). If not actively intervened, it is easy to progress to disabling stroke. This group is currently the best prevention and control window for cerebrovascular disease, and is also a hot spot in stroke research (Wang et al., 2013; Johnston et al., 2018). The MATCH trial found that combining aspirin with clopidogrel did not offer any advantage over clopidogrel alone in patients with stroke or TIAs (Diener et al., 2004). On the other hand, the CHANCE and POINT trials demonstrated that initiating dual antiplatelet therapy early in patients with minor stroke or high-risk TIAs can lower the likelihood of recurrent stroke within the first 90 days (Wang et al., 2015).

The above-mentioned and other high-quality clinical trials continue to provide clinical evidence and have rewritten the treatment recommendations of many international mainstream stroke guidelines, such as Guideline from Chinese Society of Neurology in 2014 (Wang Y. et al., 2017), 2019 Canadian Stroke Best Practice Recommendations (Boulanger et al., 2018) and guideline from American Heart Association (AHA) in 2019 (Powers et al., 2019a), confirming the efficacy and safety of short-term dual-antiplatelet therapy in patients with minor stroke in the acute phase. Although guidelines recommend dual antiplatelet therapy (DAPT), controversies persist regarding the comparative efficacy and safety profiles among different regimens (e.g., Ticagrelor/Clopidogrel/Dipyridamole plus Aspirin). Current research remains predominantly limited to single head-to-head comparisons, lacking sufficient comprehensive investigations to delineate the effectiveness of different DAPT strategies. The safety in terms of bleeding risk is also unclear, and further studies are needed to confirm the superiority and inferiority. In this study, randomized controlled trials (RCT) and high-quality retrospective studies were systematically reviewed by Bayesian network meta-analysis to comprehensively compare the efficacy and safety of different antiplatelet therapy in patients with minor stroke or TIAs, thereby providing more powerful evidence-based support for clinical decision-making.

## 2 Materials and methods

The current study follows the Preferred Reporting Items for Systematic Reviews and Meta-Analyses of Network Meta-Analyses (PRISMA-NMA) framework. The complete protocol was registered in PROSPERO with registration number CRD42024537462. Since it does not involve personal information of patients, ethical approval is not required for this study.

### 2.1 Data sources and searches

We conducted a search of the PubMed, Embase, Web of Science, and Cochrane Library databases to identify studies evaluating the effects of antiplatelet and thrombolytic therapies in minor strokes and TIAs, from inception to 12 September 2024. Only English-language publications were included. The search strategy was based on three primary concepts using the PICOS framework: (“platelet aggregation inhibitors” OR “antiplatelet” OR “antiplatelet drug”) AND (“minor stroke” OR “transient ischemic attack”) AND “human” AND “clinical trial.” Medical Subject Headings (MeSH) and related keywords were utilized to identify relevant studies. The search strategy is outlined in Supplementary Table S1. To identify additional and follow-up studies, the references of relevant articles were reviewed. The database searches were independently carried out by two authors, who removed duplicates, examined the titles and abstracts, and reviewed the full texts to select studies that met the inclusion criteria. Any differences in judgment were resolved through discussions with a third author.

### 2.2 Eligibility criteria

The following inclusion criteria were applied: 1) the study could be either a randomized or non-randomized clinical trial; 2) participants were required to be adults with a National Institutes of Health Stroke Scale (NIHSS) score of 5 or below, or individuals experiencing a high-risk transient ischemic attack (TIA), defined by an ABCD2 score of 4 or more, who received antiplatelet therapy within 72 h of symptom onset; 3) the intervention involved dual antiplatelet therapy (DAPT), consisting of aspirin combined with another antiplatelet drug such as clopidogrel, ticagrelor, prasugrel, dipyridamole, ticlopidine, or indobufen, compared to single antiplatelet therapy (SAPT) with aspirin, clopidogrel, ticagrelor, prasugrel, dipyridamole, ticlopidine, or indobufen; 4) the study had to report at least one of the clinical outcomes outlined below.

Studies were excluded based on the following criteria: 1) absence of a control group; 2) non-cohort study designs, such as cross-sectional studies or case reports; 3) studies focusing on outcomes that were not relevant to the research; and 4) studies with missing or incomplete primary data, which were unsuitable for statistical analysis or where full information could not be retrieved from the corresponding authors.

### 2.3 Data extraction

A standardized form was used to collect data from the selected studies. The information extracted included: publication year, study design, participant demographics (age and gender), sample size, treatment and control protocols, severity of stroke and TIAs, medication dosages, treatment duration, follow-up period, and outcome analyses. Two authors independently carried out the data extraction, while a third author verified the accuracy of all extracted information.

### 2.4 Clinical outcomes

The efficacy outcomes assessed included all-cause mortality, excellent outcomes (defined as a modified Rankin Scale (mRS) score of 0–1), functional independence (mRS 0–2), recurrent strokes, major vascular events, ischemic strokes, and functional disabilities (mRS 2–6). Safety outcomes encompassed any bleeding events, ranging from mild to severe, including intracerebral hemorrhage (ICH). The specific definitions of all-cause mortality, bleeding events, and major vascular events used in each study are provided in Supplementary Tables S3–S5.

### 2.5 Quality assessment

We adhered to the Cochrane Collaboration’s guidelines to assess the risk of bias in the included RCTs. We assessed each trial for seven potential biases—selection, performance, detection, attrition, reporting, and others—using the R 4.3.1 software package. For each criterion, the risk of bias was classified as “low,” “high,” or “unclear.”

We evaluated the methodological quality of the cohort studies in this analysis using the ROBINS-1 tool, which assesses the risk of bias in non-randomized intervention studies (Sterne et al., 2016). The ROB score for each observational study was evaluated across seven domains. The first three domains addressed pre- and at-intervention biases, while the remaining four focused on post-intervention biases. The overall ROB score was determined according to the ROBINS-1 guidelines. Two evaluators independently assessed each domain, and the results were recorded. Discrepancies were addressed by consulting a third evaluator for resolution.

### 2.6 Statistical analysis

We performed the Bayesian network meta-analysis (NMA) using the “BUGSnet” and “Gemtc” software packages in R (version 4.3.1). Odds ratio (OR) with 95% confidence interval (CI) was chosen as the effect size. The optimal model was chosen based on leverage plots and the deviance information criterion (DIC). In the presence of significant heterogeneity (I^2^ > 50%), we employed random model for analysis, investigated the sources of variability, and conducted sensitivity and subgroup analyses. If heterogeneity persisted despite these efforts, the results were presented using a descriptive analytical approach. In the case of sparse data, the powerful flexibility of Bayesian methods can be used to reduce the error. Specifically, this is achieved by incorporating prior assumptions into the model through the prior distribution, allowing the model to make reasonable inferences even in the presence of sparse data. Additionally, by performing multiple samplings of the model’s posterior distribution, we can better estimate the uncertainty of the parameters, thereby making the model more stable and reliable and providing more robust inferences.

To assess convergence and model stability, we used trace and density plots, along with the potential scale reduction factor (PSRF). For closed loops, we evaluated the assumption of transitivity by comparing direct and indirect evidence, applying the node-splitting method for consistency analysis. A P-value greater than 0.05 was considered indicative of strong consistency, whereas a P-value less than 0.05 indicated inconsistency. To assess the ranking probabilities of the interventions, we utilized the surface under the cumulative ranking curve (SUCRA), where 0% represented the least favorable outcome and 100% represented the most favorable. If more than 10 studies were included, publication bias was assessed.

## 3 Results

### 3.1 Study selection

**FIGURE 1:**
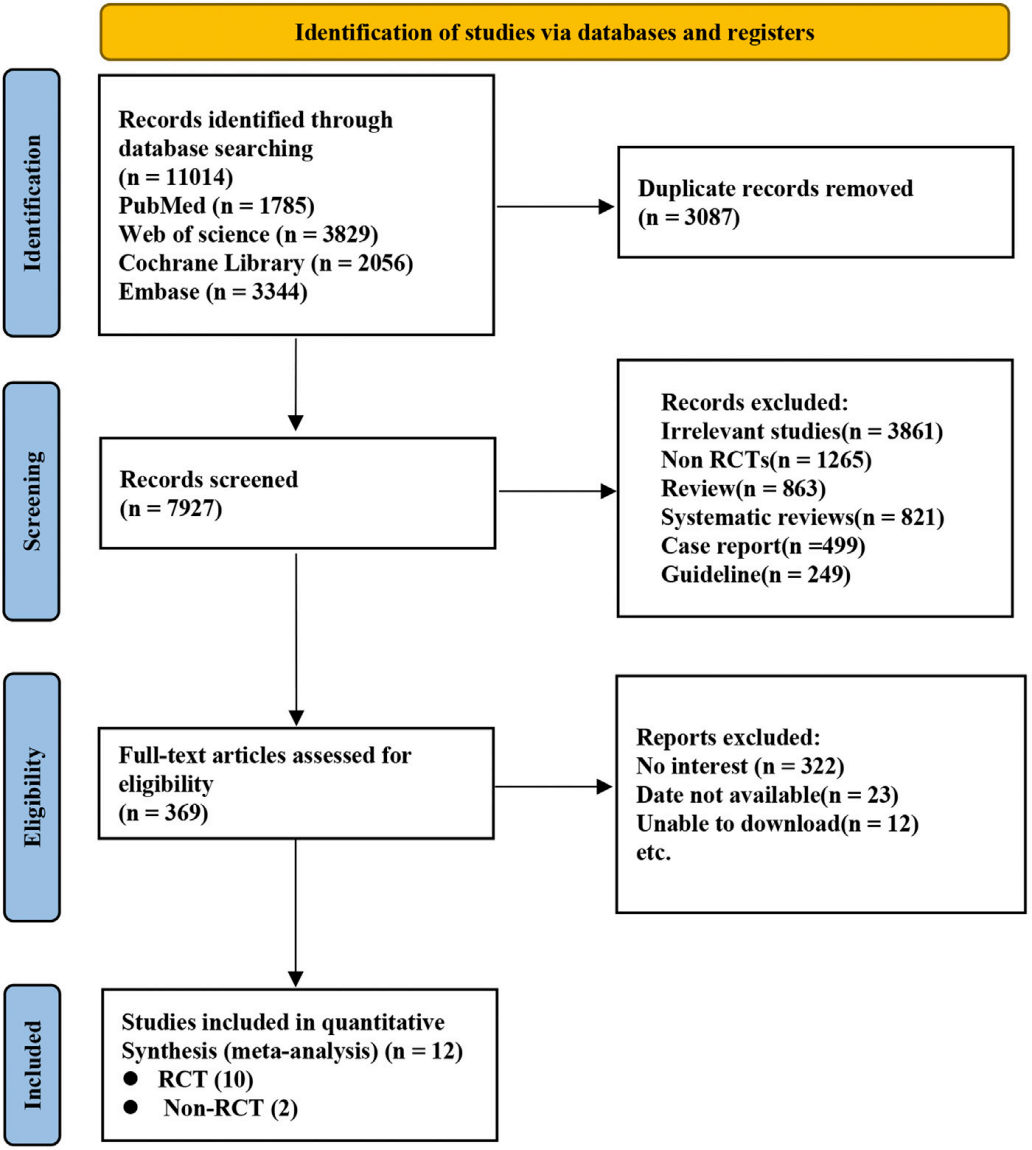
A flow diagram for selection of patients in this study.

A total of 11,014 records were initially retrieved from electronic databases through an extensive search. After excluding 3,087 duplicates and reviewing the titles and abstracts of the remaining studies, 7,927 were excluded. Ultimately, Ten RCTs (Wang et al., 2013; Johnston et al., 2018; Gao et al., 2023; Halkes et al., 2006; He et al., 2015; Johnston et al., 2016; Johnston et al., 2020; Kennedy et al., 2007; Wang et al., 2019; Wang et al., 2021) and two Non-RCTs (Huang et al., 2024; Kim et al., 2019) were selected for inclusion in this analysis (Figure 1).

### 3.2 Characteristics of the included trials

A total of 10 RCTs and 2 Non-RCTs involving 61,281 patients with minor stroke or TIAs were included in the analysis. The participants were categorized into six treatment groups based on the regimen they received: SAPTs (Aspirin and Ticagrelor) and DAPTs (Clopidogrel + Aspirin, Cilostazol + Aspirin, Dipyridamole + Aspirin, and Ticagrelor + Aspirin). Of the 61,281 patients, 52.1% were male and 47.9% female, with ages ranging from 61.5 to 71 years. The studies were conducted across multiple countries, including China, the United States, Australia, and others, and were published between 2006 and 2023 (Supplementary Table S2). The networks of treatment comparisons for both efficacy and safety outcomes are presented in Supplementary Figures S1, S2.

### 3.3 Risk of bias

As depicted in Supplementary Figure S3, the Cochrane risk of bias assessment tool was utilized to evaluate the methodological rigor and potential biases in the 10 RCTs included in this study. Two studies raised concerns regarding the concealment of group assignment and the blinding of both participants and staff. However, for other aspects, the studies were deemed to have a minimal risk of bias, indicating that their overall quality was high. In Supplementary Figure S4, among the two ROBIN studies, one was found to have some concerns regarding the risk of bias. There may be uncertain selection bias, therefore it is assessed as “unclear”.

### 3.4 All-cause mortality

**FIGURE 2:**
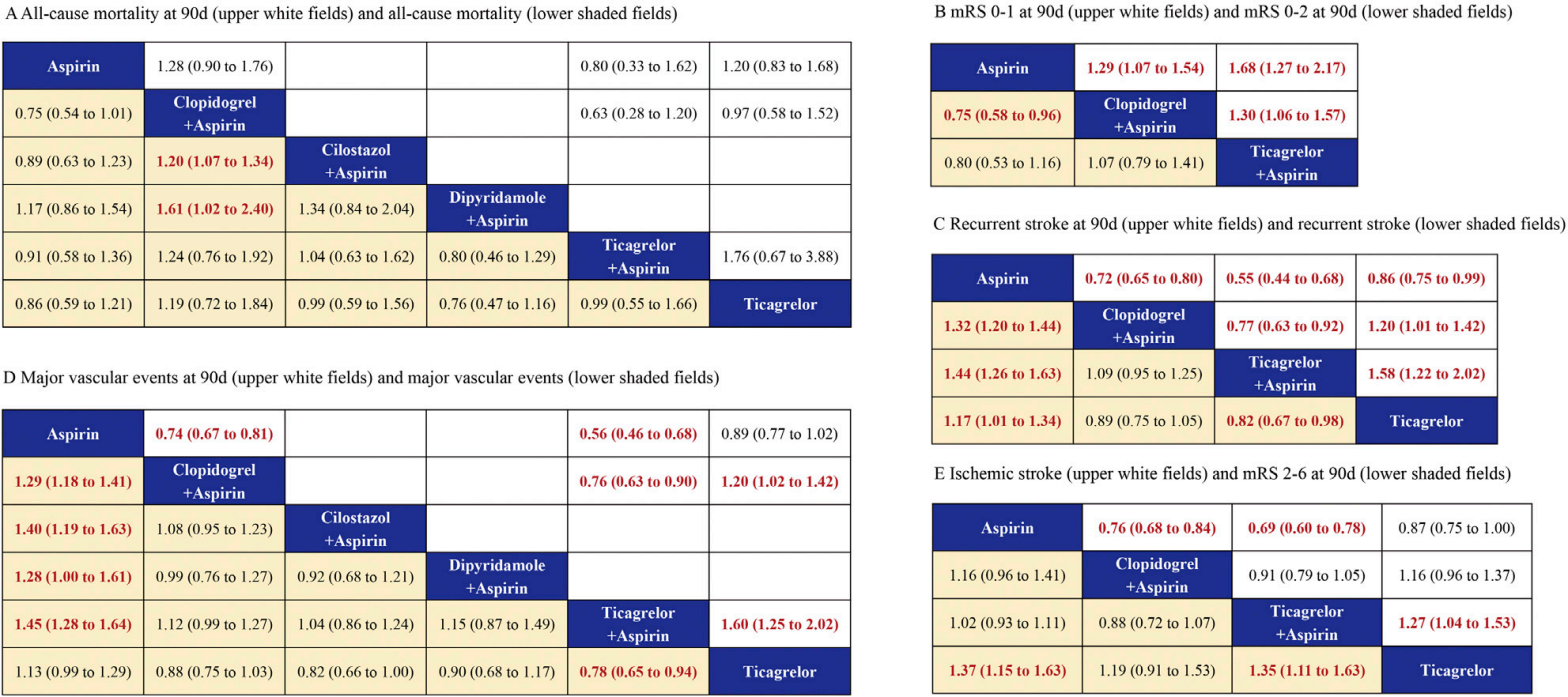
Summary of the efficacy outcomes of network meta-analysis in minor strokes or TIAs. The effect sizes are presented as odds ratio of the means with 95% credible intervals. The diagram should be read from left to right with OR<1 favoring the column-defining treatment and this meant that the treatment in the column was associated with a lower risk for the outcome than the treatment in the row. The significant results are presented in red bold fonts. mRS, modified rankin scale. **(A)** All-cause monality at 90 days (uppe.r white fields) and all-cause monality (lower shaded fields). **(B)** mRS 0-1 at 90 days (oppeJ white fields) aJld mRS 0-2 at 90 days (lower shaded fields). **(C)** Recurrent stroke at 90 days (upper wltite fields) and recurrent stroke (lower shaded fields). **(D)** Major vascular events at 90 days (upper white fields) and major vascular events ( lower shaded fields). **(E)** Ischemic stroke (upper whire fields) and mRS 2-6 at 90 days (lower shaded fields).

**FIGURE 3:**
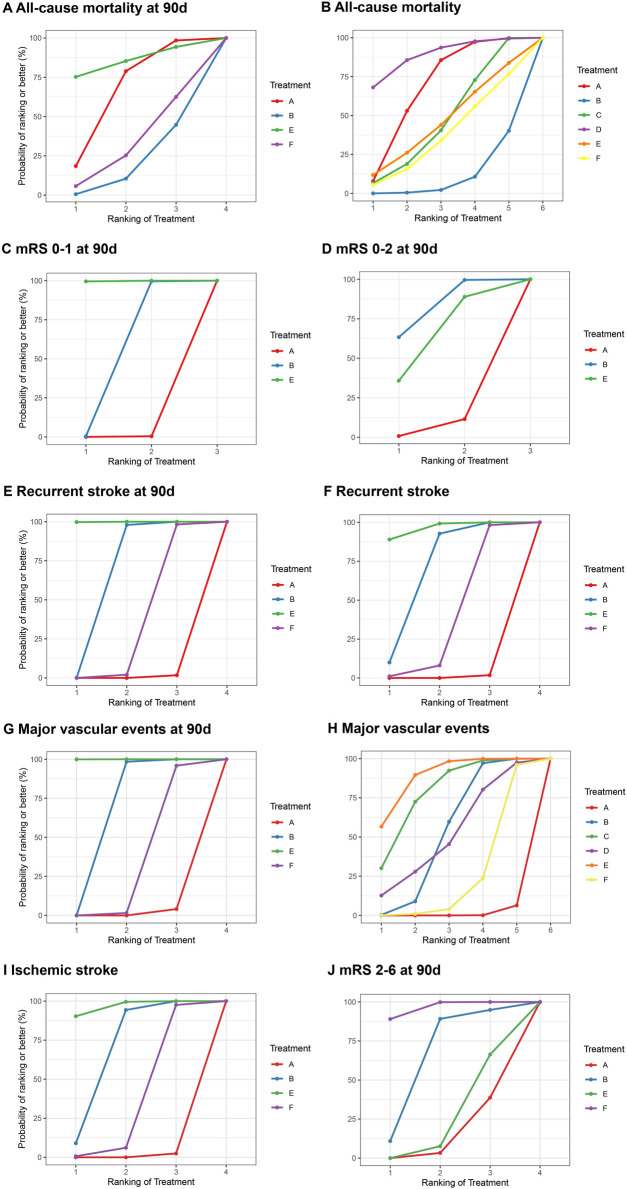
Summary of the efficacy outcomes of network meta-analysis in minor strokes. The effect sizes are presented as odds ratio of the means with 95% credible intervals. The diagram should be read from left to right with OR<1 favoring the column-defining treatment and this meant that the treatment in the column was associated with a lower risk for the outcome than the treatment in the row. The significant results are presented in red bold fonts. DAPT, dual antiplatelet therapy; IVT, intravenous thrombolysis; mRS, modified Rankin scale; SAPT, single antiplatelet therapy. **(A)** All-cause mortality at 90 days. **(B)** All use mortality. **(C)** mRS 0-1al 90 days. **(D)** mRS 0-2 al 90 days. **(E)** Recurrent stroke at 90 days. **(F)** Recurrent stroke. **(G)** Major vascular events at 90 days. **(H)** Major vascular events. **(I)** lschemlc stroke. **(J)** mRS 2-6 at 90 days.

A total of 10 studies provided data on all-cause mortality, involving 60,239 patients, with the evidence network shown in Supplementary Figures S1A, S1B. The treatments administered facilitated both direct and indirect comparisons, thereby partially closing the loop, as depicted in the figures. Compared with Clopidogrel + Aspirin, Cilostazol + Aspirin and Dipyridamole + Aspirin were significantly decreased in all-cause mortality during follow-up (Figure 2A). Dipyridamole + Aspirin was associated with the lowest risk of all-cause mortality during follow-up, followed by Aspirin, Cilostazol + Aspirin, Ticagrelor + Aspirin, Ticagrelor and Clopidogrel + Aspirin (Figures 3A, B).

### 3.5 Excellent outcome (mRS 0–1)

2 included studies with 12,512 participants accounted for excellent outcomes (mRS 0–1) at 90 days. For each intervention drug, direct and indirect comparisons were established (Supplementary Figure S1C). Compared to Aspirin and Clopidogrel + Aspirin, Ticagrelor + Aspirin (OR = 1.68; 95% CI 1.27–2.17, OR = 1.30; 95% CI 1.06–1.57) was associated with a significant difference in excellent outcome (mRS 0–1) at 90 days. Compared to Aspirin, Clopidogrel + Aspirin (OR = 1.29; 95% CI 1.07–1.54) was associated with a significant difference in excellent outcome (mRS 0–1) at 90 days (Figure 2B). Ticagrelor + Aspirin was associated with the highest occurrence of excellent outcomes (mRS 0–1) at 90 days, followed by Clopidogrel + Aspirin and Aspirin (Figure 3C).

### 3.6 Functional independence (mRS 0–2)

One included study with 12,512 participants accounted for excellent outcomes (mRS 0–2) at 90 days. For each intervention drug, direct and indirect comparisons were established (Supplementary Figure S1D). Compared to Clopidogrel + Aspirin, Aspirin (OR = 0.75; 95% CI 0.58–0.96) was associated with a significant difference in excellent outcome (mRS 0–2) at 90 days (Figure 2D). These results indicated that Clopidogrel + Aspirin was the most effective regimen in improving the functional independence (mRS 0–2) of patients at 90 days with the biggest SUCRA, followed by Ticagrelor + Aspirin and Aspirin (Figure 3D).

### 3.7 Recurrent stroke

9 studies provided data on stroke occurrences, involving a total of 51,053 patients. The evidence relationship diagram in Supplementary Figures S1E, S1F illustrates the network of evidence. The interventions used enabled both direct and indirect comparisons, helping to partially close the loop. In comparison with Aspirin, Clopidogrel + Aspirin (OR = 0.72; 95% CI 0.65–0.80), Ticagrelor + Aspirin (OR = 0.55; 95% CI 0.44–0.86) and Ticagrelor (OR = 0.86; 95% CI 0.75–0.99) were significantly associated with lower stroke recurrence at 90 days (Figure 2C). In comparison with Clopidogrel + Aspirin and Ticagrelor + Aspirin, Ticagrelor (OR = 1.20; 95% CI 1.01–1.42, OR = 1.58; 95% CI 1.22–2.02) were significantly associated with higher stroke recurrence at 90 days (Figure 2C). Compared to Clopidogrel + Aspirin, Ticagrelor + Aspirin (OR = 0.77; 95% CI 0.63–0.92) was significantly associated with lower stroke recurrence at 90 days (Figure 2C). Aspirin was significantly associated with higher stroke recurrence during follow-up, compared with Clopidogrel + Aspirin (OR = 1.32; 95% CI 1.20–1.44), Ticagrelor + Aspirin (OR = 1.44; 95% CI 1.26–1.63) and Ticagrelor (OR = 1.17; 95% CI 1.01–1.34) (Figure 2C). Analytical results of SUCRA cumulative probability revealed that Ticagrelor + Aspirin had the lowest number of stroke recurrence, followed by Clopidogrel + Aspirin, Ticagrelor and Aspirin (Figures 3E, F).

### 3.8 Major vascular events

11 studies involving 6 different interventions and a combined participant population of 60,631 were included. The treatments applied allowed for both direct and indirect comparisons (Supplementary Figures S1G, S1H). In comparison with Aspirin and Ticagrelor, Clopidogrel + Aspirin and Ticagrelor + Aspirin were significantly associated with lower major vascular events at 90 days (Figure 2D). Compared to Clopidogrel + Aspirin, Ticagrelor + Aspirin (OR = 0.76; 95% CI 0.63–0.90) was significantly associated with lower major vascular events at 90 days (Figure 2D). Aspirin was significantly associated with higher major vascular events during follow-up, compared with Clopidogrel + Aspirin, Cilostazol + Aspirin, Dipyridamole + Aspirin and Ticagrelor + Aspirin (Figure 2D). The SUCRA analysis cumulative probability revealed that Ticagrelor + Aspirin had the lowest number of major vascular events, followed by Cilostazol + Aspirin, Clopidogrel + Aspirin, Dipyridamole + Aspirin, Ticagrelor and Aspirin (Figures 3G, H).

### 3.9 Ischemic stroke

In minor strokes or TIAs, Ischemic stroke was reported in 7 studies involving 4 different interventions, encompassing 47,453 patients who collectively experienced 3,168 events. The drugs used for intervention facilitated both direct and indirect comparisons, helping to partially complete the loop (Supplementary Figure S1I). Compared to Aspirin, Clopidogrel + Aspirin (OR = 0.76; 95% CI 0.68–0.84) and Ticagrelor + Aspirin (OR = 0.69; 95% CI 0.60–0.78) was significantly associated with lower ischemic stroke during follow-up (Figure 2E). Ticagrelor + Aspirin was associated with the lowest occurrence of ischemic stroke, followed by Clopidogrel + Aspirin, Ticagrelor and Aspirin (Figure 3I).

### 3.10 Functional disability (mRS 2–6)

Functional disability (mRS 2–6) at 90 days were reported in 4 studies that enrolled 36,727 patients incurring a combined total of 3,548 events. The drugs administered allowed for both direct and indirect comparisons (Supplementary Figure S1J). In comparison with Ticagrelor, Aspirin (OR = 1.37; 95% CI 1.15–1.63) and Ticagrelor + Aspirin (OR = 1.35; 95% CI 1.11–1.63) was significantly associated with higher functional disability (mRS 2–6) at 90 days (Figure 3E). The results of SUCRA analysis showed that the cumulative probability with Ticagrelor had the lowest functional disability (mRS 2–6) at 90 days, followed by Clopidogrel + Aspirin, Ticagrelor + Aspirin and Aspirin (Figure 3J).

### 3.11 Any type of bleeding events

**FIGURE 4:**
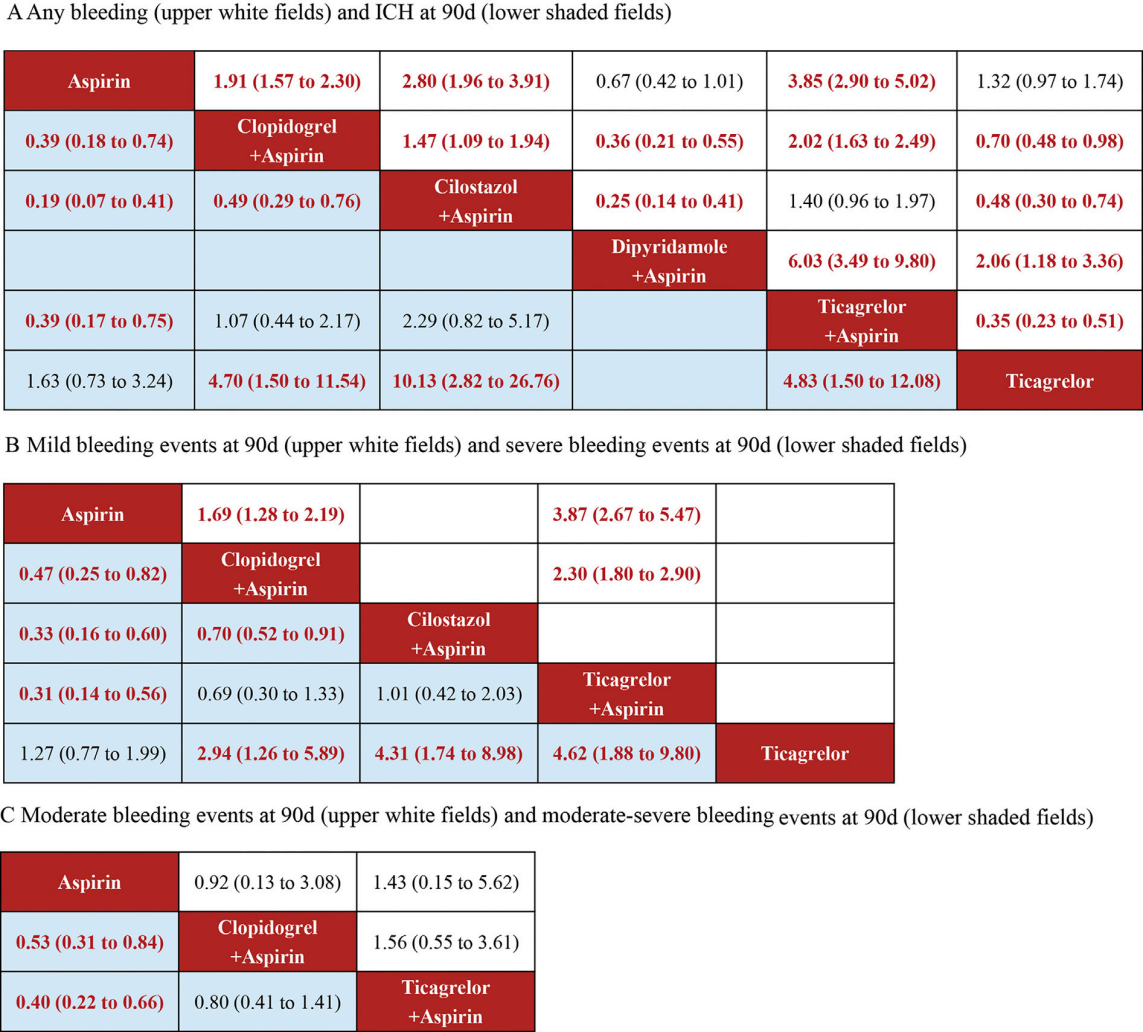
Summary of the safety outcomes of network meta-analysis in minor strokes or TIAs. The effect sizes are presented as the odds ratio of means with 95% credible intervals. The diagram should be read from left to right with OR<1 favoring the column-defining treatment and this meant that the treatment in the column was associated with a lower risk for the outcome than the treatment in the row. The significant results are presented in red bold fonts. ICH, intracerebral hemorrhage; sICH, symptomatic intracerebral hemorrhage. **(A)** Any bleeding (uppe white fields) and CH at 90 days (lower shaded fields). **(B)** Mild bleeding events at 90 days (upper white fields) and severe bleeding events at 90 days (lower shaded fields). **(C)** Moderate bleeding events at 90 days (upper white fields) and moderate-sewre bleeding events at 90 days (lower shaded fields).

**FIGURE 5:**
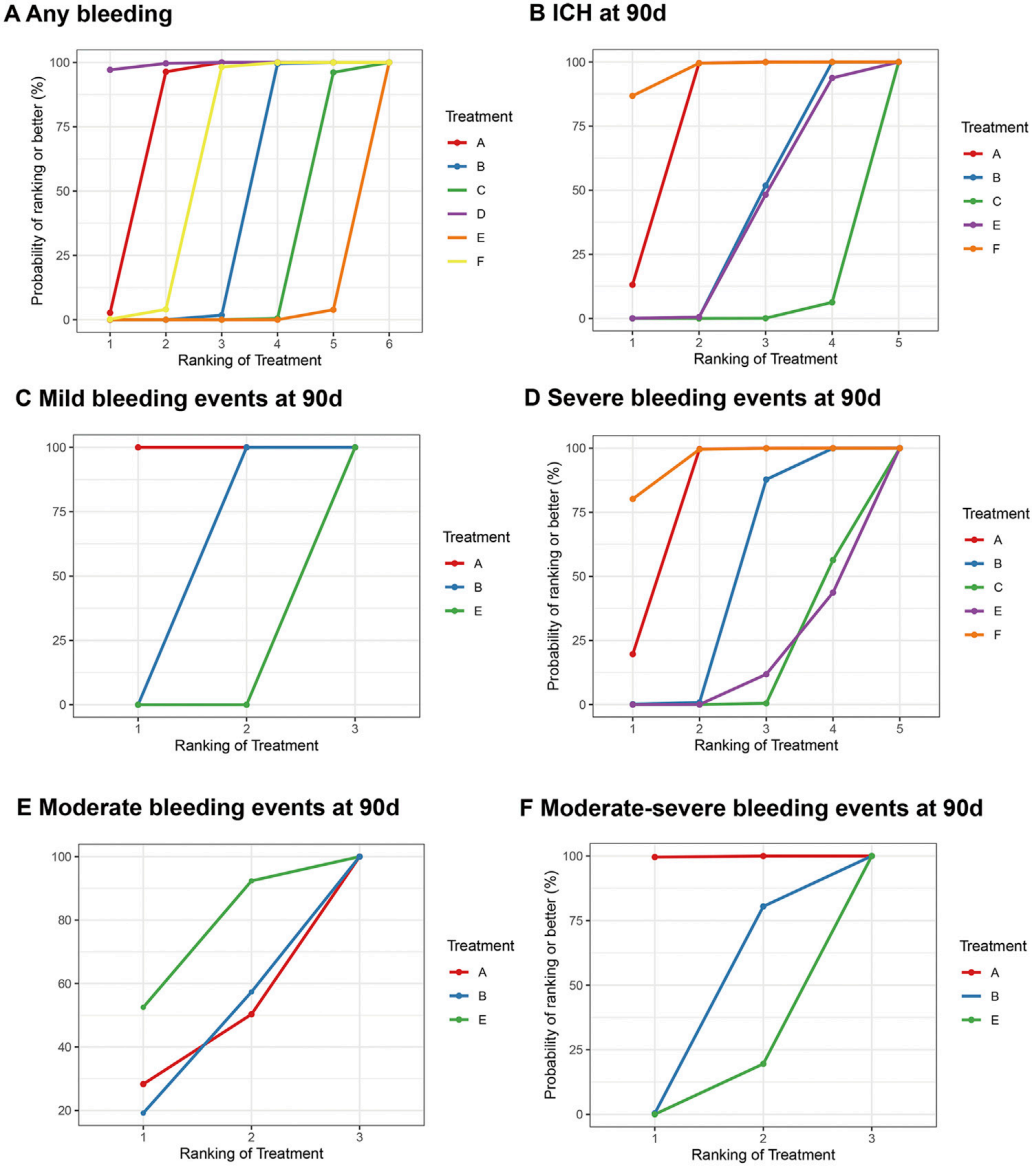
Summary of the safety outcomes of network meta-analysis in minor strokes. The effect sizes are presented as the odds ratio of means with 95% credible intervals. The diagram should be read from left to right with OR<1 favoring the column-defining treatment and this meant that the treatment in the column was associated with a lower risk for the outcome than the treatment in the row. The significant results are presented in red bold fonts. DAPT, dual antiplatelet therapy; ICH, intracerebral hemorrhage; IVT, intravenous thrombolysis; SAPT, single antiplatelet therapy; sICH, symptomatic intracerebral hemorrhage. **(A)** Any b eeding. **(B)** ICH at 90 days. **(C)** Mild bleeding events at 90 days. **(D)** Severe bleeding events at 90 days. **(E)** Moderate bleeding events at 90 days. **(F)** Moderate-severe bleeding events at 90 days.

11 studies reported bleeding events during follow-up, and these included 58,070 participants. The drugs involved enabled both direct and indirect comparisons, contributing to the partial closure of the loop (Supplementary Figure S2A). The estimated effects indicated that Dipyridamole + Aspirin was significantly associated with a lower bleeding risk, when compared with other interventions except Aspirin (Figure 4A). And Ticagrelor + Aspirin was significantly associated with a higher bleeding risk, when compared with other interventions except Cilostazol + Aspirin (Figure 4A). Compared to Aspirin and Ticagrelor, the risk of bleeding was significantly increased in Clopidogrel + Aspirin and Cilostazol + Aspirin (Figure 4A). The SUCRA cumulative probability revealed that Dipyridamole + Aspirin had the lowest bleeding risk, followed by Aspirin, Ticagrelor, Clopidogrel + Aspirin, Cilostazol + Aspirin and Ticagrelor + Aspirin (Figure 5A).

### 3.12 ICH

In minor strokes or TIAs, 6 RCTs and 1 Non-RCTs occurrence of ICH at 90 days, encompassing 44,635 patients who collectively experienced 179 events. For each intervention drug used, both direct and indirect comparisons were conducted (Supplementary Figure S2B). The estimated effects indicated that Aspirin and Ticagrelor were significantly associated with a lower ICH risk at 90 days, compared with Clopidogrel + Aspirin, Cilostazol + Aspirin and Ticagrelor + Aspirin (Figure 4A). Compared to Cilostazol + Aspirin, the risk of bleeding was significantly reduced in Clopidogrel + Aspirin (OR = 0.49; 95% CI 0.29–0.76) (Figure 4A). The SUCRA cumulative probability results revealed that Ticagrelor had the lowest ICH at 90 days, followed by Aspirin, Clopidogrel + Aspirin, Ticagrelor + Aspirin and Cilostazol + Aspirin (Figure 5B).

### 3.13 Mild bleeding events

In minor strokes or TIAs, 6 studies accounted for mild bleeding events observed at 90 days with 23,885 participants included in the study. Compared to Clopidogrel + Aspirin and Ticagrelor + Aspirin, the risk of mild bleeding events at 90 days was significantly reduced in Aspirin (Figure 4B). Compared to Ticagrelor + Aspirin, the risk of mild bleeding events at 90 days was significantly reduced in Clopidogrel + Aspirin (Figure 4B). The SUCRA cumulative probability results revealed that Aspirin had the lowest mild bleeding events at 90 days, followed by Clopidogrel + Aspirin and Ticagrelor + Aspirin (Figure 5C).

### 3.14 Severe bleeding events

In minor strokes or TIAs, 7 studies accounted for severe bleeding events at 90 days and involved 48,192 participants who were included in the NMA. Each intervention drug was subjected to both direct and indirect comparisons (Supplementary Figure S2D). When compared to Aspirin and Ticagrelor, the risk of severe bleeding at 90 days was significantly increased in patients undergoing Clopidogrel + Aspirin, Cilostazol + Aspirin and Ticagrelor + Aspirin (Figure 4B). Compared to Cilostazol + Aspirin, the risk of severe bleeding events at 90 days was significantly reduced in Clopidogrel + Aspirin (Figure 4B). The SUCRA cumulative probability results revealed that Ticagrelor had the lowest severe bleeding risk at 90 days, followed by Aspirin, Clopidogrel + Aspirin, Cilostazol + Aspirin and Ticagrelor + Aspirin (Figure 5D).

### 3.15 Moderate bleeding events

In minor strokes or TIAs, only 2 RCTs reported moderate bleeding events at 90 days. The estimated effects indicated that there was no significant difference in moderate bleeding events at 90 days with three interventions (Figure 4C). The SUCRA cumulative probability results revealed that Aspirin had the lowest moderate bleeding risk at 90 days, followed by Clopidogrel + Aspirin and Ticagrelor + Aspirin (Figure 5E).

### 3.16 Moderate-severe bleeding events

In minor strokes or TIAs, 4 studies reported moderate-severe bleeding events at 90 days. Each intervention drug underwent both direct and indirect comparisons (Supplementary Figure S2G). Compared to Aspirin, the risk of moderate-severe bleeding at 90 days was significantly increased in Clopidogrel + Aspirin and Ticagrelor + Aspirin (Figure 4C). The SUCRA cumulative probability results revealed that Aspirin had the lowest Moderate-severe bleeding risk at 90 days, followed by Clopidogrel + Aspirin and Ticagrelor + Aspirin (Figure 5F).

### 3.17 Assessment of efficacy and safety

**FIGURE 6:**
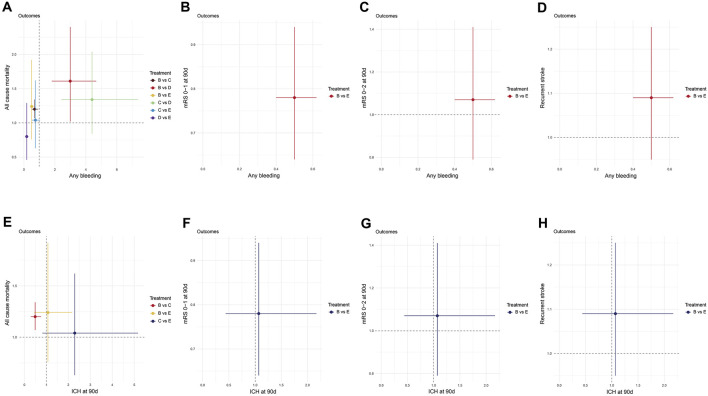
Two-dimension Forest plot for efficacy and safety outcomes. Effect sizes are presented as odds ratio of means with 95% credible interval. Individual treatments are represented by different colored nodes. **(A)** All-cause mortality-Any bleeding; **(B)** mRS 0–1 at 90 days-Any bleeding; **(C)** mRS 0–2 at 90 days-Any bleeding; **(D)** Recurrent stroke-Any bleeding; **(E)** All-cause mortality-ICH at 90days; **(F)** mRS 0–1 at 90 days-ICH at 90days; **(G)** mRS 0–2 at 90 days-ICH at 90days; **(H)** Recurrent stroke-ICH at 90 days. **(A)** Aspirin; **(B)** Clopidogrel + Aspirin; **(C)** Cilostazol + Aspirin; **(D)** Dipyridamole + Aspirin; **(E)** Ticagrelor + Aspirin; **(F)** Ticagrelor. mRS, modified rankin scale.

The results of this study suggest that the efficacy of DAPT is significantly better than that of SAPT, but the risk of bleeding is higher. Therefore, in order to more intuitively display the efficacy and safety results between different DAPTs, we drew a two-dimensional forest plot. The results suggested that compared to Clopidogrel + Aspirin, Dipyridamole + Aspirin was associated with lower all-cause mortality and lower risk of any bleeding (Figure 6A). Compared to Cilostazol + Aspirin, Dipyridamole + Aspirin significantly reduced the risk of any bleeding, but there was no significant difference in improving all-cause mortality (Figure 6A). Clopidogrel + Aspirin and Dipyridamole + Aspirin significantly reduced the risk of any bleeding compared with Ticagrelor + Aspirin, but there was no significant difference in improving all-cause mortality (Figure 6A). Based on the above results, if only the risk of all-cause mortality and any bleeding is considered, Dipyridamole + Aspirin seems to be the optimal DAPT.

Clopidogrel + Aspirin significantly reduced the risk of any bleeding, compared with Ticagrelor + Aspirin, but there was no significant difference in the risk of ICH at 90 days. Compared to Clopidogrel + Aspirin, Ticagrelor + Aspirin did not differ significantly in improving all-cause mortality, stroke recurrence and functional independence (mRS 0–2), but significantly improved neurological function (mRS 0–1) (Figures 6B–H).

### 3.18 Network consistency and heterogeneity

To assess the influence of Non-RCTs on the meta-analysis outcomes, a subgroup analysis was performed based on RCT and Non-RCTs studies. The results indicated that the meta-analysis findings were robust, as illustrated in Supplementary Figures S5, S6. For all outcomes, the evaluation of the cumulative posterior residual deviance and the number of unconstrained data points in both fixed-effects (FE) and random-effects (RE) network meta-analyses (NMAs) suggested that the model fit was appropriate. Heterogeneity analysis revealed that most comparisons across the included studies exhibited low to moderate heterogeneity, and the DIC comparisons showed no significant differences between the FE and RE consistency models (Supplementary Figures S7, S8). To ensure the reliability of the results, the RE model was applied in all meta-analyses. DIC comparisons and leverage plot assessments did not suggest any violations of the consistency assumption. Detailed model fit statistics and consistency checks are provided in Supplementary Figure S9 through 14.

### 3.19 Publication bias

To evaluate the potential presence of publication bias and the impact of small-study effects, we used a comparison-adjusted funnel plot. The approximately symmetrical shape of the funnel plot indicated no evidence of publication bias (Supplementary Figures S15, S16). It is essential to recognize, however, that the accuracy of using funnel plots to evaluate publication bias may be compromised for clinical outcomes, especially when the number of included studies is limited.

## 4 Discussion

This systematic review and NMA included data from 10 RCTs and 2 Non-RCTs involving 61,281 minor stroke or TIAs patients. We found that DAPT was significantly more effective than aspirin in preventing post-stroke neurological dysfunctions (mRS 0–1 and mRS 0–2), recurrent stroke, major vascular events, and ischemic strokes for up to 90 days. However, compared to Ticagrelor, Clopidogrel + Aspirin and Ticagrelor + Aspirin significantly reduced the risk of recurrent stroke at 90 days and major vascular events at 90 days. Cilostazol + aspirin and Dipyridamole + Aspirin have no significant advantage in efficacy compared with Ticagrelor, which may be due to the lack of high-quality clinical studies or the lack of statistical difference. In comparison with Ticagrelor, Aspirin was significantly associated with higher risk of recurrent stroke and functional disability (mRS 2–6) at 90 days. The NMA results of different DAPT suggested that Ticagrelor + Aspirin was significantly more effective than Clopidogrel + Aspirin in preventing post-stroke neurological dysfunctions (mRS 0–1), recurrent stroke and major vascular events for up to 90 days. In addition, we also found that compared with Clopidogrel + Aspirin, Cilostazol + Aspirin and Dipyridamole + Aspirin can significantly reduce all-cause mortality during follow-up.

It is worth noting that DAPT may be more effective than SAPT, but the risk of bleeding is also an important factor that we must consider. Most safety results suggested that Aspirin and Ticagrelor were associated with a lower risk of bleeding compared with DAPT. But the risk of any bleeding in Dipyridamole + Aspirin is not significantly different from that in Aspirin, and is even significantly lower than in Ticagrelor. In addition, compared with Clopidogrel + Aspirin, Ticagrelor + Aspirin is associated with an increased risk of any bleeding and mild bleeding at 90 days, and there is no significant difference in other bleeding risks.

In our network meta-analysis (NMA), we included 12 studies comparing dual antiplatelet therapy (DAPT) and single antiplatelet therapy (SAPT) in both intervention and control groups. Among these, nine studies used Clopidogrel + Aspirin and three used Ticagrelor + Aspirin as DAPT regimens, while two studies used Cilostazol + Aspirin and Dipyridamole + Aspirin. According to current international guidelines for minor stroke and TIAs, the American Heart Association/American Stroke Association (AHA/ASA) (Powers et al., 2019b; Dawn et al., 2021) recommends Clopidogrel + Aspirin for 21 days, which has shown significant efficacy in reducing the risk of recurrent ischemic events within 90 days following symptom onset. However, Ticagrelor is not recommended as an empirical treatment. The current guidelines rarely mention the applicability of Ticagrelor + Aspirin due to insufficient evidence, and the relevant evidence still needs to be further improved. Importantly, this study demonstrated through multiple efficacy outcomes that Ticagrelor + Aspirin was superior to Clopidogrel + Aspirin, not only in promoting neurological improvement (mRS 0–1), but also in reducing the risk of further recurrence within 90 days after symptom onset. Nevertheless, Ticagrelor + Aspirin significantly increased the risk of bleeding compared with Clopidogrel + Aspirin. The findings are expected to provide guidelines with additional evidence to support the use of Ticagrelor + Aspirin. In addition, our meta-analysis demonstrated that DAPT was more effective than SAPT in preventing stroke and improving neurological function. However, DAPT was associated with a higher safety risk compared to SAPT. These findings were consistent with the results from previous RCT meta-analyses, thereby enhancing the reliability of our results. Notably, studies with larger sample sizes likely contributed more to the overall meta-analysis findings. The four studies with the largest sample sizes in this analysis, namely, THALES (Johnston et al., 2020), INSPIRES (Gao et al., 2023), CHANCE (Wang et al., 2013) and POINT (Johnston et al., 2018), had a substantial influence on the efficacy and safety results. However, aside from the CHANCE (Wang et al., 2013) trial, differences in the DAPT loading doses used in the other three studies likely contributed to the results observed in the meta-analysis. Specifically, the THALES (Johnston et al., 2020) trial, study administered aspirin at doses of 300–325 mg, the INSPIRES (Gao et al., 2023) study used 100–300 mg aspirin, and the POINT (Johnston et al., 2018), study involved a 600 mg loading dose of clopidogrel. These dosing variations may have amplified the efficacy of DAPT and increased the risk of bleeding. Interestingly, the risk of any bleeding in Dipyridamole + Aspirin is not significantly different from that in Aspirin, and is even significantly lower than in Ticagrelor. At the same time, compared with other DAPTs, Dipyridamole + Aspirin had no significant difference in the risk of all-cause mortality and major vascular events during follow-up. It can be inferred that Dipyridamole + Aspirin may become the most promising DAPT regimen. However, among the 12 studies included in the NMA, only one studied the intervention method Dipyridamole + Aspirin, and the comparison results with other DAPT were all from indirect comparisons. Given these concerns, the findings should be approached with caution, and further high-quality research is required to validate the effectiveness of Dipyridamole + Aspirin in minor strokes or TIAs.

High-quality clinical trials have rewritten the treatment recommendations of many international mainstream stroke guidelines and confirmed the efficacy and safety of short-term DAPT in the acute phase of minor strokes and TIAs (Wang Y. et al., 2017; Boulanger et al., 2018; Powers et al., 2019a). Existing high quality clinical studies have shown that the DAPT regimen with the most sufficient clinical evidence for minor strokes or TIAs is Clopidogrel + Aspirin, followed by Ticagrelor + Aspirin. We all know that clopidogrel and ticagrelor are both P2Y12 receptor antagonists, but the metabolic pathway of ticagrelor is different from that of clopidogrel. It is mainly metabolized by the CYP3A4 enzyme and does not involve the CYP2C19 gene. Therefore, it is not affected by the polymorphism of this gene (Brilakis et al., 2013). However, clopidogrel is a prodrug that requires conversion into its active metabolite through the action of hepatic cytochrome P450 enzymes (CYP). Clopidogrel has a limited effectiveness in secondary stroke prevention in individuals carrying the CYP2C19 loss-of-function allele, which affects approximately 25% of Caucasian and 60% of Asian (Wang et al., 2016; Pan et al., 2017). To explore whether ticagrelor can replace clopidogrel and achieve better therapeutic effects, two RCTs conducted in China conducted a head-to-head comparison of Clopidogrel + Aspirin and Ticagrelor + Aspirin (Wang et al., 2019; Wang et al., 2021). The current meta-analysis suggests that Ticagrelor + Aspirin was significantly more effective than Clopidogrel + Aspirin in preventing post-stroke neurological dysfunctions (mRS 0–1), recurrent stroke and major vascular events for up to 90 days. But compared with Clopidogrel + Aspirin, Ticagrelor + Aspirin is associated with an increased risk of any bleeding and mild bleeding at 90 days, and there is no significant difference in other bleeding risks. The results of the direct comparison between Clopidogrel + Aspirin and Ticagrelor + Aspirin in this study mainly came from the two RCTs mentioned above that were conducted in China. One of the RCTs with a larger sample size included minor strokes or TIAs with CYP2C19 loss-of-function alleles. And this study undoubtedly made a greater contribution to the meta-analysis. Therefore, for minor strokes or TIAs with a lower risk of bleeding or CYP2C19 loss-of-function alleles, Ticagrelor + Aspirin may be a better choice than Clopidogrel + Aspirin.

This study has several limitations. First, the pooled data may have inherent bias due to the inclusion of both RCTs and Non-RCTs. Specifically, we included two Non-RCTs, one of which may have been subject to selection bias (Huang et al., 2024). To avoid selection bias during the analysis, employed a new-user design with an active comparator, as done extensively in previous pharmacoepidemiology studies (Yoshida et al., 2015), this helps reduce potential bias, but some selection bias may still exist, For example, several lifestyle-related factors (e.g., smoking, alcohol consumption, and body mass index) might confound results of the included study. Therefore, although efforts were made in this study to minimize selection bias, as an observational study, it still carries potential biases, as the choice of whether patients received SAPT or DAPT was not randomized. This may affect the robustness of the NMA results of this study, which is also our limitation. Second, there were variations across the included studies regarding the timing of treatment initiation, baseline NIHSS scores, definitions of minor stroke, and anti-platelet dosing protocols. These differences could have influenced the outcome assessment in the meta-analysis. Third, some outcome indicators in the network diagram lacked closed loops, which suggests that there may be inconsistencies across the studies and analytical results. Fourth, due to the limited research on Dipyridamole + Aspirin (only 1 study), most of which demonstrates its efficacy and safety advantages through indirect comparisons, it is difficult to adequately support an evaluation of its greater benefits, making it difficult to draw definitive conclusions from the results. Lastly, certain outcome subsets included fewer than 10 studies, which may limit the validity of the findings for these outcomes, even though publication bias analysis indicated low risk. Therefore, the results should be interpreted with caution, as potential confounders must be taken into account. However, many of our conclusions were robust and well-supported, and this novel study offers valuable insights for future interventions in the treatment of minor strokes or TIAs.

## 5 Conclusion

For minor strokes or TIAs with a low bleeding risk or CYP2C19 loss-of-function alleles, Ticagrelor + Aspirin may be a better choice than Clopidogrel + Aspirin. Due to limited studies, the superiority of Dipyridamole + Aspirin is still difficult to conclude, and further high-quality studies are needed to verify the benefits of Dipyridamole + Aspirin in minor stroke or TIAs.

## Data Availability

The original contributions presented in the study are included in the article/Supplementary Material, further inquiries can be directed to the corresponding authors.
