# Supplementary material for: Ticagrelor or dipyridamole plus aspirin may be a promising antiplatelet therapy in patients with minor stroke or transient ischemic attack: a bayesian network meta-analysis
Source: Front Pharmacol. 2025 Mar 26;16:1561564. doi: 10.3389/fphar.2025.1561564 (PMC11978618; doi:10.3389/fphar.2025.1561564)
Supplement: Supplementary file 1 [file DataSheet1.docx]

Supplementary Material

**Supplemental Figure 1.** Network plots for the efficacy outcomes.

**Supplemental Figure 2.** Network plots for the safety outcomes.

**Supplemental Figure 3.** Risk of bias of randomized clinical trials.

**Supplemental Figure 4.** Risk of bias of non-randomized clinical trials.

**Supplemental Figure 5.** Forest plots for the network meta-analysis of secondary efficacy outcomes.

**Supplemental Figure 6.** Forest plots for the network meta-analysis of secondary safty outcomes.

**Supplemental Figure 7.** Heterogeneity of results for efficacy outcomes.

**Supplemental Figure 8.** Heterogeneity of results for safety outcomes.

**Supplemental Figure 9.** Leverage plot of efficacy outcomes.

**Supplemental Figure 10.** Leverage plot of safety outcomes.

**Supplemental Figure 11.** The trace and density plots of safety outcomes.

**Supplemental Figure 12.** The trace and density plots of efficacy outcomes.

**Supplemental Figure 13.** Nodes-splitting analysis of efficacy outcomes.

**Supplemental Figure 14.** Nodes-splitting analysis of safety outcomes.

**Supplemental Figure 15.** Publication Bias Assessment of efficacy outcomes.

**Supplemental Figure 16.** Publication Bias Assessment of safety outcomes.

**Supplemental Table 1.** Search Strategy

**Supplemental Table 2.** Characteristics of the included trials

**Supplemental Table 3.** All-cause mortality outcomes in each study

**Supplemental Table 4.** Bleeding definitions

**Supplemental Table 5.** Major vascular events definitions

**Supplemental Table 1. Search Strategy**

| **PubMed** **(September 12, 2024)** | | |
| --- | --- | --- |
| **#1** | (((((((((((((((((((((((((((Ischemic Strokes[MeSH Terms])) OR Ischemic Stroke*[Title/Abstract]) OR (Stroke, Ischemic[Title/Abstract])) OR (Ischaemic Stroke[Title/Abstract])) OR (Ischaemic Strokes[Title/Abstract])) OR (Stroke, Ischaemic[Title/Abstract])) OR (Cryptogenic Ischemic Stroke[Title/Abstract])) OR (Cryptogenic Ischemic Strokes[Title/Abstract])) OR (Ischemic Stroke, Cryptogenic[Title/Abstract])) OR (Stroke, Cryptogenic Ischemic[Title/Abstract])) OR (Cryptogenic Stroke[Title/Abstract])) OR (Cryptogenic Strokes[Title/Abstract])) OR (Stroke, Cryptogenic[Title/Abstract])) OR (Cryptogenic Embolism Stroke[Title/Abstract])) OR (Cryptogenic Embolism Strokes[Title/Abstract])) OR (Embolism Stroke, Cryptogenic[Title/Abstract])) OR (Stroke, Cryptogenic Embolism[Title/Abstract])) OR (Wake-up Stroke[Title/Abstract])) OR (Stroke, Wake-up[Title/Abstract])) OR (Wake up Stroke[Title/Abstract])) OR (Wake-up Strokes[Title/Abstract])) OR (Acute Ischemic Stroke[Title/Abstract])) OR (Acute Ischemic Strokes[Title/Abstract])) OR (Ischemic Stroke, Acute[Title/Abstract])) OR (Stroke, Acute Ischemic[Title/Abstract])) OR (cerebral arterial thrombosis[Title/Abstract])) OR (ischemic cerebral apoplexy[Title/Abstract]) | 86,939 |
| **#2** | (((((((((((((((((((((((("Platelet Aggregation Inhibitors"[Mesh]) OR (Platelet Antiaggregant[Title/Abstract])) OR (Blood Platelet Aggregation Inhibitor[Title/Abstract])) OR (Blood Platelet Antiaggregant[Title/Abstract])) OR (Platelet Aggregation Inhibitor[Title/Abstract])) OR (Platelet Antiaggregants[Title/Abstract])) OR (Platelet Inhibitor[Title/Abstract])) OR (Antiplatelet Agent[Title/Abstract])) OR (Antiplatelet Drug[Title/Abstract])) OR (Blood Platelet Antagonist[Title/Abstract])) OR (Platelet Antagonist[Title/Abstract])) OR ((("Aspirin"[Mesh]) OR (Aspirin[Title/Abstract])) OR (Acetylsalicylic Acid[Title/Abstract]))) OR ("Clopidogrel"[Mesh])) OR (Clopidogrel[Title/Abstract])) OR (P2Y12 inhibitors[Title/Abstract])) OR ("Ticagrelor"[Mesh])) OR (Ticagrelor[Title/Abstract])) OR (Brilique[Title/Abstract])) OR (Brilinta[Title/Abstract])) OR ("Cilostazol"[Mesh])) OR (Cilostazol[Title/Abstract])) OR (Pletal[Title/Abstract])) OR (Indobufen[Title/Abstract])) OR ("Prasugrel Hydrochloride"[Mesh])) OR (Prasugrel[Title/Abstract]) | 110,019 |
| **#3** | (((((((randomized controlled trial[Publication Type]) OR (controlled clinical trial[Publication Type])) OR (randomized[Title/Abstract])) OR (placebo[Title/Abstract])) OR ("Clinical Trials as Topic"[Mesh:NoExp])) OR (randomly[Title/Abstract])) OR (trial[Title/Abstract])) NOT (("Animals"[Mesh]) NOT ("Humans"[Mesh])) | 1,749,651 |
| **#4** | #1 AND #2 AND #3 | 1,785 |

| **Web of science (September 12, 2024)** | | |
| --- | --- | --- |
| **#1** | ((((((((((((((((((((((((((TS=(Ischemic Strokes)) OR TS=(Stroke, Ischemic)) OR TS=(Ischaemic Stroke)) OR TS=(Ischaemic Strokes)) OR TS=(Stroke, Ischaemic)) OR TS=(Cryptogenic Ischemic Stroke)) OR TS=(Cryptogenic Ischemic Strokes)) OR TS=(Ischemic Stroke, Cryptogenic)) OR TS=(Stroke, Cryptogenic Ischemic)) OR TS=(Cryptogenic Stroke)) OR TS=(Cryptogenic Strokes)) OR TS=(Stroke, Cryptogenic)) OR TS=(Cryptogenic Embolism Stroke)) OR TS=(Cryptogenic Embolism Strokes)) OR TS=(Embolism Stroke, Cryptogenic)) OR TS=(Stroke, Cryptogenic Embolism)) OR TS=(Wake-up Stroke)) OR TS=(Stroke, Wake-up)) OR TS=(Wake up Stroke)) OR TS=(Wake-up Strokes)) OR TS=(Acute Ischemic Stroke)) OR TS=(Acute Ischemic Strokes)) OR TS=(Ischemic Stroke, Acute)) OR TS=(Stroke, Acute Ischemic)) OR TS=(cerebral arterial thrombosis)) OR TS=(ischemic cerebral apoplexy)) | 141,775 |
| **#2** | (((((((((((((((((((((((TS=(Platelet Aggregation Inhibitors)) OR TS=(Platelet Antiaggregant)) OR TS=(Blood Platelet Aggregation Inhibitor)) OR TS=(Blood Platelet Antiaggregant)) OR TS=(Platelet Aggregation Inhibitor)) OR TS=(Platelet Antiaggregants)) OR TS=(Platelet Inhibitor)) OR TS=(Antiplatelet Agent)) OR TS=(Antiplatelet Drug)) OR TS=(Blood Platelet Antagonist)) OR TS=(Platelet Antagonist)) OR TS=(Aspirin)) OR TS=(Acetylsalicylic Acid)) OR TS=(Clopidogrel)) OR TS=(P2Y12 inhibitors)) OR TS=(Ticagrelor)) OR TS=(Brilique)) OR TS=(Brilinta)) OR TS=(Cilostazol)) OR TS=(Pletal)) OR TS=(Indobufen)) OR TS=(Prasugrel Hydrochloride)) OR TS=(Prasugrel)) | 108,281 |
| **#3** | ((((((TS=(randomized controlled trial)) OR TS=(controlled clinical trial)) OR TS=(randomized)) OR TS=(placebo)) OR TS=(randomly)) OR TS=(trial)) | 2,225,972 |
| **#4** | (TS=(Animals)) NOT TS=(Humans) | 785,247 |
| **#5** | #3 NOT #4 | 2148,394 |
| **#6** | #1 AND #2 AND #5 | 3,829 |
| **Embase (September 12, 2024)** | | |
| **#1** | 'ischemic stroke'/exp | 37,989 |
| **#2** | 'ischemic strokes':ab,kw,ti OR 'ischemic stroke':ab,kw,ti OR 'stroke, ischemic':ab,kw,ti OR 'ischaemic stroke':ab,kw,ti OR 'ischaemic strokes':ab,kw,ti OR 'stroke, ischaemic':ab,kw,ti OR 'cryptogenic ischemic stroke':ab,kw,ti OR 'cryptogenic ischemic strokes':ab,kw,ti OR 'ischemic stroke, cryptogenic':ab,kw,ti OR 'stroke, cryptogenic ischemic':ab,kw,ti OR 'cryptogenic stroke':ab,kw,ti OR 'cryptogenic strokes':ab,kw,ti OR 'stroke, cryptogenic':ab,kw,ti OR 'cryptogenic embolism stroke':ab,kw,ti OR 'cryptogenic embolism strokes':ab,kw,ti OR 'embolism stroke, cryptogenic':ab,kw,ti OR 'stroke, cryptogenic embolism':ab,kw,ti OR 'wake-up stroke':ab,kw,ti OR 'stroke, wake-up':ab,kw,ti OR 'wake up stroke':ab,kw,ti OR 'wake-up strokes':ab,kw,ti OR 'acute ischemic stroke':ab,kw,ti OR 'acute ischemic strokes':ab,kw,ti OR 'ischemic stroke, acute':ab,kw,ti OR 'stroke, acute ischemic':ab,kw,ti OR 'cerebral arterial thrombosis':ab,kw,ti OR 'ischemic cerebral apoplexy':ab,kw,ti | 144,574 |
| **#3** | #1 OR #2 | 152,986 |
| **#4** | 'Platelet Aggregation Inhibitors':ab,kw,ti OR 'Platelet Antiaggregant':ab,kw,ti OR 'Blood Platelet Aggregation Inhibitor':ab,kw,ti OR 'Blood Platelet Antiaggregant':ab,kw,ti OR 'Platelet Aggregation Inhibitor':ab,kw,ti OR 'Platelet Antiaggregants':ab,kw,ti OR 'Platelet Inhibitor':ab,kw,ti OR 'Antiplatelet Agent':ab,kw,ti OR 'Antiplatelet Drug':ab,kw,ti OR 'Blood Platelet Antagonist':ab,kw,ti OR 'Platelet Antagonist':ab,kw,ti | 8,384 |
| **#5** | 'acetylsalicylic acid'/exp | 267,284 |
| **#6** | '2 acetoxybenzoate':ab,kw,ti OR '2 acetoxybenzoic acid':ab,kw,ti OR 'aspirin':ab,kw,ti OR 'aspirina':ab,kw,ti OR 'acetylsalicylic acid':ab,kw,ti | 103,397 |
| **#7** | 'clopidogrel'/exp | 80,065 |
| **#8** | 'p2y12 inhibitors':ab,kw,ti OR 'iscover':ab,kw,ti OR 'clopidogrel':ab,kw,ti | 30,026 |
| **#9** | 'ticagrelor'/exp | 16,845 |
| **#10** | 'brilinta':ab,kw,ti OR 'brilique':ab,kw,ti OR 'possia':ab,kw,ti OR 'ticagrelor':ab,kw,ti | 7,879 |
| **#11** | 'cilostazol'/exp | 7,779 |
| **#12** | 'pletaal':ab,kw,ti OR 'pletal':ab,kw,ti OR 'cilostazol':ab,kw,ti | 3,356 |
| **#13** | 'indobufen'/exp | 571 |
| **#14** | 'ibustrin':ab,kw,ti OR 'indobufen':ab,kw,ti | 247 |
| **#15** | 'prasugrel'/exp | 12,569 |
| **#16** | 'prasugrel benzenesulfonate':ab,kw,ti OR 'prasugrel besilate':ab,kw,ti OR 'prasugrel besylate':ab,kw,ti OR 'prasugrel hcl':ab,kw,ti OR 'prasugrel hydrochloride':ab,kw,ti OR 'prasugrel':ab,kw,ti | 5,438 |
| **#17** | #4 OR #5 OR #6 OR #7 OR #8 OR #9 OR #10 OR #11 OR #12 OR #13 OR #14 OR #15 OR #16 | 306,686 |
| **#18** | 'randomized controlled trial'/exp | 853,850 |
| **#19** | 'controlled clinical trial'/exp | 1036,110 |
| **#20** | 'randomization'/exp | 100,372 |
| **#21** | 'placebo'/exp | 426,688 |
| **#22** | 'randomized controlled trial':ti,ab,kw OR 'rct':ti,ab,kw OR 'controlled clinical trial':ti,ab,kw OR 'random allocation':ti,ab,kw OR 'placebos':ti,ab,kw OR 'random':ti,ab,kw OR 'randomization':ti,ab,kw OR 'trial':ti,ab,kw | 1,654,130 |
| **#23** | 'animals':ti,ab,kw NOT 'humans':ti,ab,kw | 896,183 |
| **#24** | (#18 OR #19 OR #20 OR #21 OR #22) NOT #23 | 2,497,414 |
| **#25** | #3 AND #17 AND #24 | 3,344 |
| **Cochranne (September 12, 2024)** | | |
| **#1** | MeSH descriptor: [Ischemic Stroke] explode all trees | 1,704 |
| **#2** | ("Ischaemic Stroke" OR " Stroke, Ischaemic" OR " Ischaemic Strokes" OR " Stroke, Ischemic" OR " Ischemic Strokes" OR " Wake up Stroke" OR " Wake-up Strokes" OR " Wake-up Stroke" OR " Stroke, Wake-up" OR " Cryptogenic Strokes" OR " Cryptogenic Embolism Stroke" OR " Stroke, Cryptogenic Embolism" OR " Stroke, Cryptogenic" OR " Cryptogenic Embolism Strokes" OR " Ischemic Stroke, Cryptogenic" OR " Cryptogenic Ischemic Strokes" OR " Cryptogenic Stroke" OR " Stroke, Cryptogenic Ischemic" OR " Embolism Stroke, Cryptogenic" OR " Cryptogenic Ischemic Stroke" OR " Acute Ischemic Stroke" OR " Stroke, Acute Ischemic" OR " Acute Ischemic Strokes" OR " Ischemic Stroke, Acute" OR " cerebral arterial thrombosis " OR " ischemic cerebral apoplexy " OR "Ischemic Stroke"):ti,ab,kw | 15,194 |
| **#3** | #1 OR #2 | 15,258 |
| **#4** | MeSH descriptor: [Platelet Aggregation Inhibitors] explode all trees | 5,976 |
| **#5** | ("Platelet Aggregation Inhibitors" OR "Blood Platelet Antagonist" OR "Platelet Antagonists" OR "Antagonist, Blood Platelet" OR "Platelet Antagonist, Blood" OR "Blood Platelet Antagonists" OR "Antagonist, Platelet" OR "Platelet Antagonist" OR " Platelet Inhibitors" OR "Platelet Inhibitor" OR "Inhibitor, Platelet" OR "Platelet Antiaggregant" OR "Antiaggregant, Platelet" OR "Platelet Aggregation Inhibitor" OR "Blood Platelet Aggregation Inhibitor" OR "Blood Platelet Antiaggregant" OR "Aggregation Inhibitor, Platelet" OR "Blood Platelet Aggregation Inhibitors" OR "Antiaggregants, Platelet" OR "Antiaggregant, Blood Platelet" OR "Inhibitor, Platelet Aggregation" OR "Platelet Antiaggregants" OR "Blood Platelet Antiaggregants" OR "Platelet Antiaggregant, Blood" OR "Antiplatelet Agents" OR "Agent, Antiplatelet" OR "Antiplatelet Drugs" OR "Drug, Antiplatelet" OR "Antiplatelet Drug" OR "Antiplatelet Agent"):ti,ab,kw | 7,609 |
| **#6** | MeSH descriptor: [Aspirin] explode all trees | 8,057 |
| **#7** | ("Aspirin" OR "Acid, Acetylsalicylic" OR "Easprin" OR "Acetylsalicylic Acid" OR "2 acetoxybenzoate" OR "2 acetoxybenzoic acid"):ti,ab,kw | 19,039 |
| **#8** | MeSH descriptor: [Clopidogrel] explode all trees | 2,761 |
| **#9** | ("Plavix" OR "Iscover" OR "p2y12 inhibitors" OR "clopidogrel"):ti,ab,kw | 6,619 |
| **#10** | MeSH descriptor: [Ticagrelor] explode all trees | 1,224 |
| **#11** | ("Brilique" OR "Brilinta" OR "ticagrelor"):ti,ab,kw | 2,467 |
| **#12** | MeSH descriptor: [Cilostazol] explode all trees | 463 |
| **#13** | ("pletaal" OR "pletal" OR "cilostazol"):ti,ab,kw | 978 |
| **#14** | ("ibustrin" OR "indobufen"):ti,ab,kw | 117 |
| **#15** | MeSH descriptor: [Prasugrel Hydrochloride] explode all trees | 632 |
| **#16** | ("Effient" OR "Efient; Prasugrel" OR "Hydrochloride, Prasugre" OR "Prasugrel Hydrochloride" OR "prasugrel benzenesulfonate" OR "prasugrel besilate" OR "prasugrel besylate"):ti,ab,kw | 665 |
| **#17** | #4 OR #5 OR #6 OR #7 OR #8 OR #9 OR #10 OR #11 OR #12 OR #13 OR #14 OR #15 OR #16 | 25,136 |
| **#18** | MeSH descriptor: [Randomized Controlled Trial] explode all trees | 27,103 |
| **#19** | MeSH descriptor: [Controlled Clinical Trial] explode all trees | 41,256 |
| **#20** | MeSH descriptor: [Random Allocation] explode all trees | 26,032 |
| **#21** | MeSH descriptor: [Placebos] explode all trees | 27,242 |
| **#22** | ("Randomized Controlled Trial " OR "RCT" OR " Controlled Clinical Trial " OR " Random Allocation " OR " Placebos " OR " Random " OR " Randomization " OR " Trial"):ti,ab,kw | 1,220,170 |
| **#23** | ("Animals" NOT "Humans"):ti,ab,kw | 5,231 |
| **#24** | (#18 OR #19 OR #20 OR #21 OR #22) NOT #23 | 1,224,957 |
| **#25** | #3 AND #17 AND #24 | 2,056 |

**
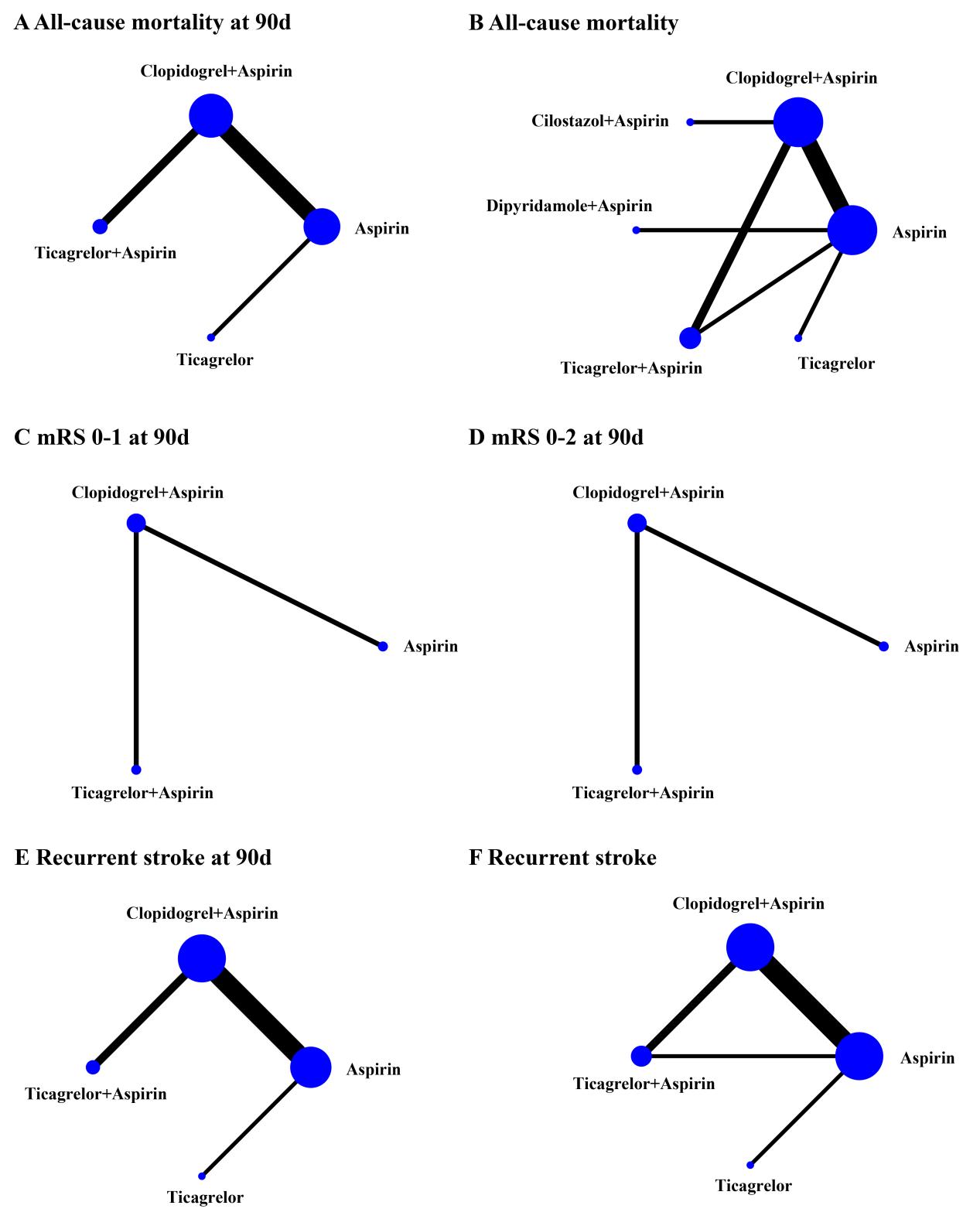
**

**
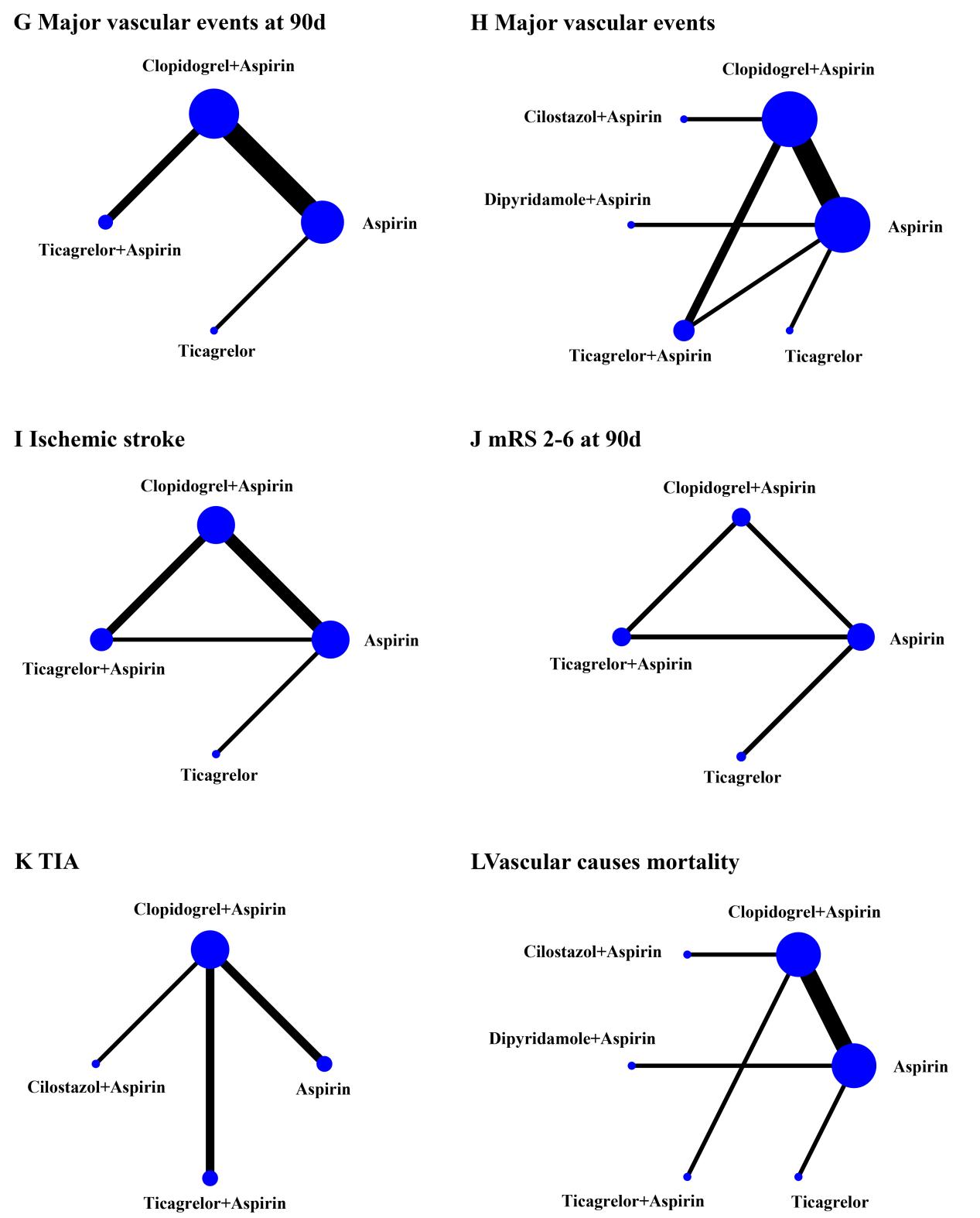
**

**Supplemental Figure 1.** Network plots for the efficacy outcomes. The nodes represent the treatments to be compared, and the straight lines represent the direct comparisons. The size of the nodes and between nodes line thickness is proportional to the sample size and research quantity. mRS, modified rankin scale.


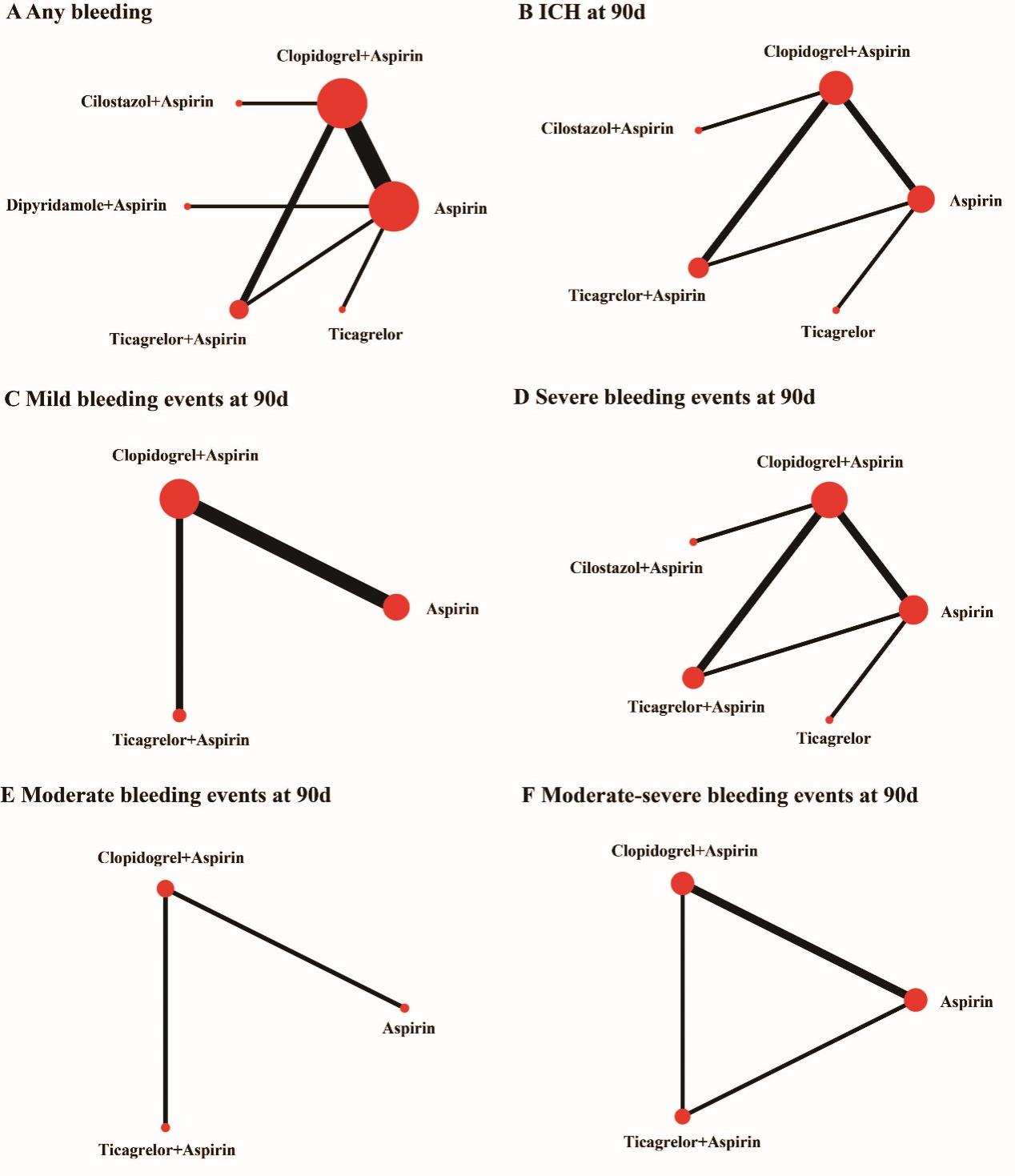


**Supplemental Figure 2.** Network plots for the safety outcomes. The nodes represent the treatments to be compared, and the straight lines represent the direct comparisons. The size of the nodes and between nodes line thickness is proportional to the sample size and research quantity. ICH, intracerebral hemorrhage.


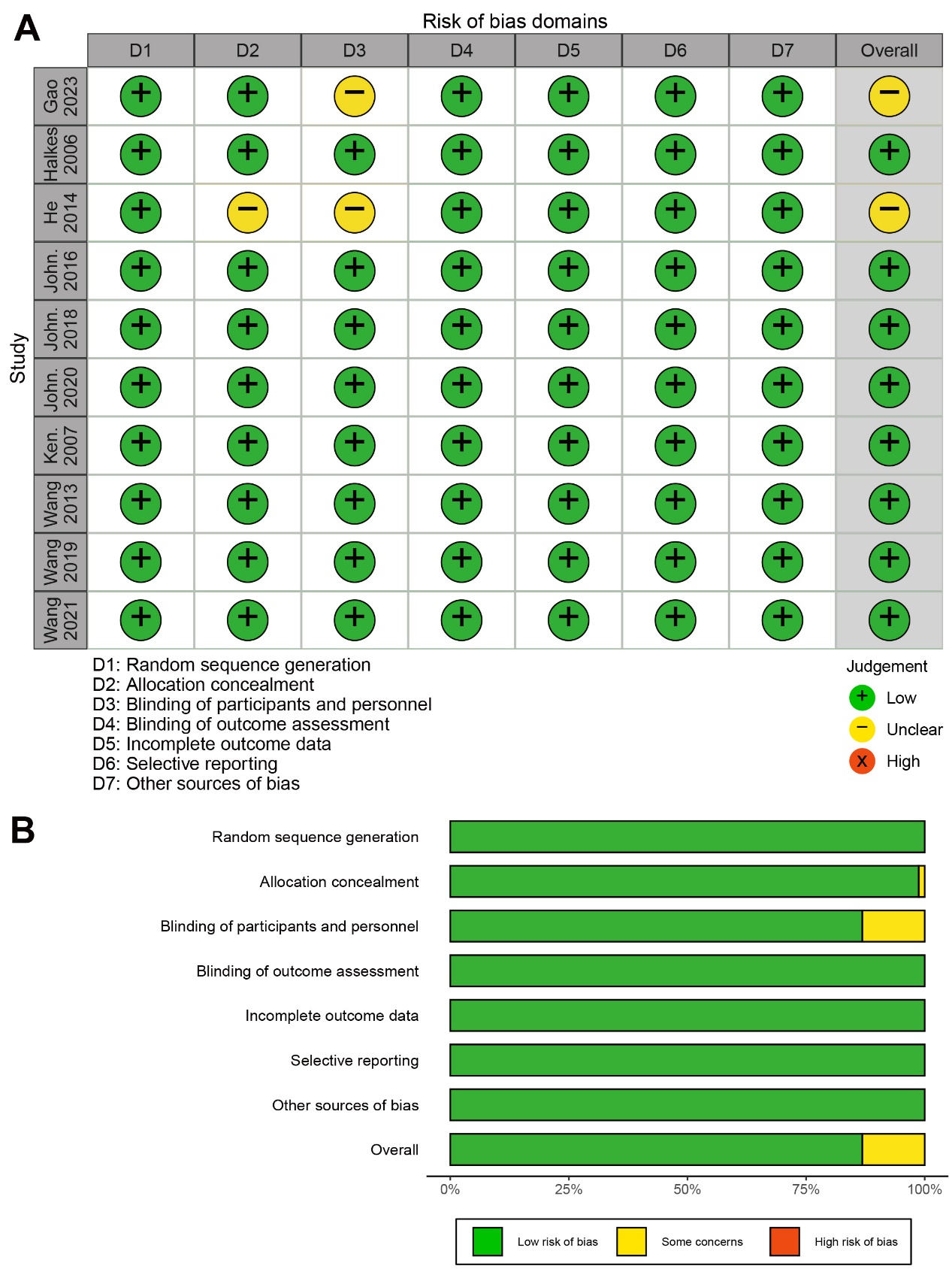


**Supplemental Figure 3.** Risk of bias of randomized clinical trials. (A) Risk of bias summary: judgments about each bias item for each study; (B) Risk of bias summary graph.


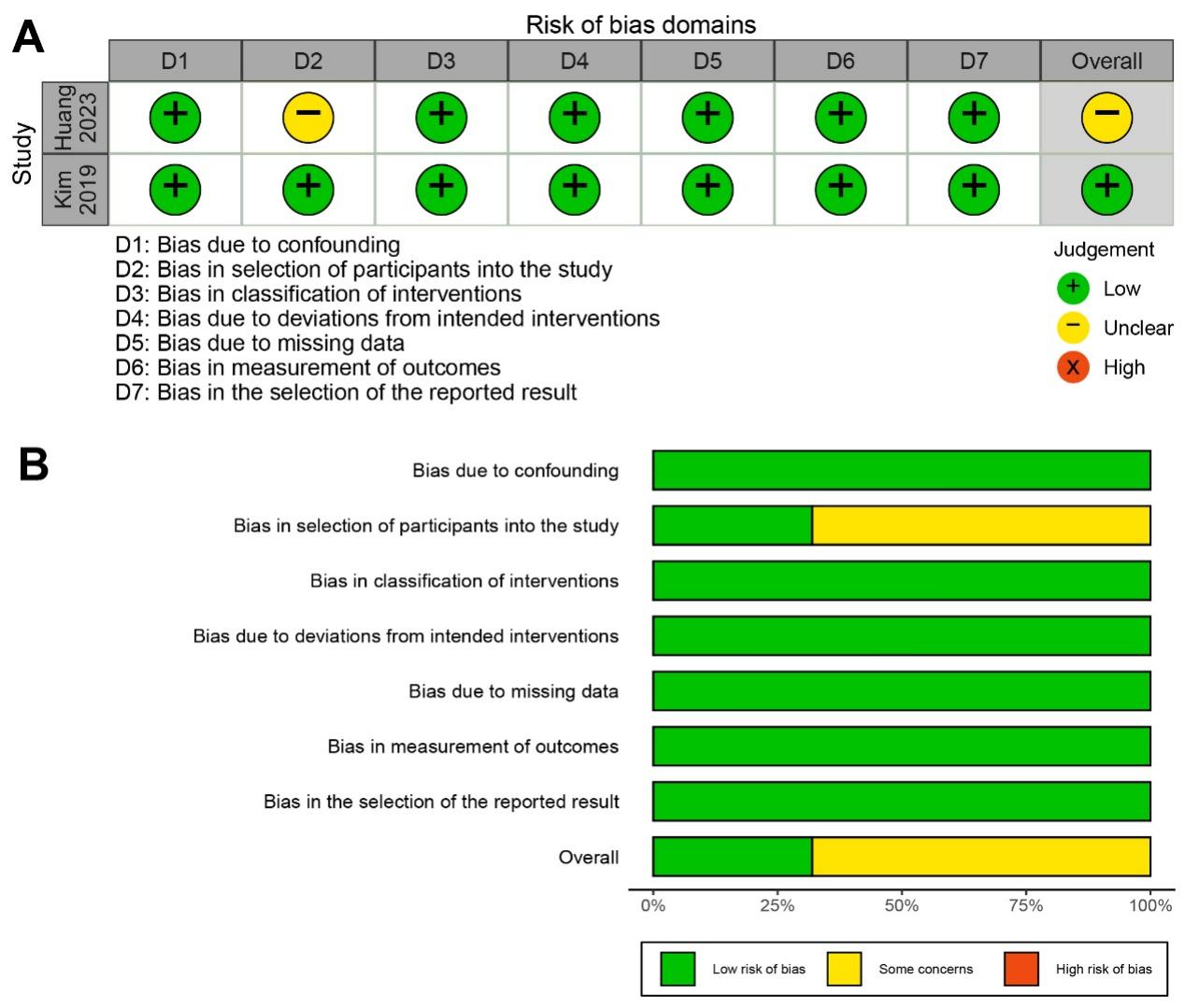


**Supplemental Figure 4.** Risk of bias of non-randomized clinical trials. (A) Risk of bias summary: judgments about each bias item for each study; (B) Risk of bias summary graph.

**
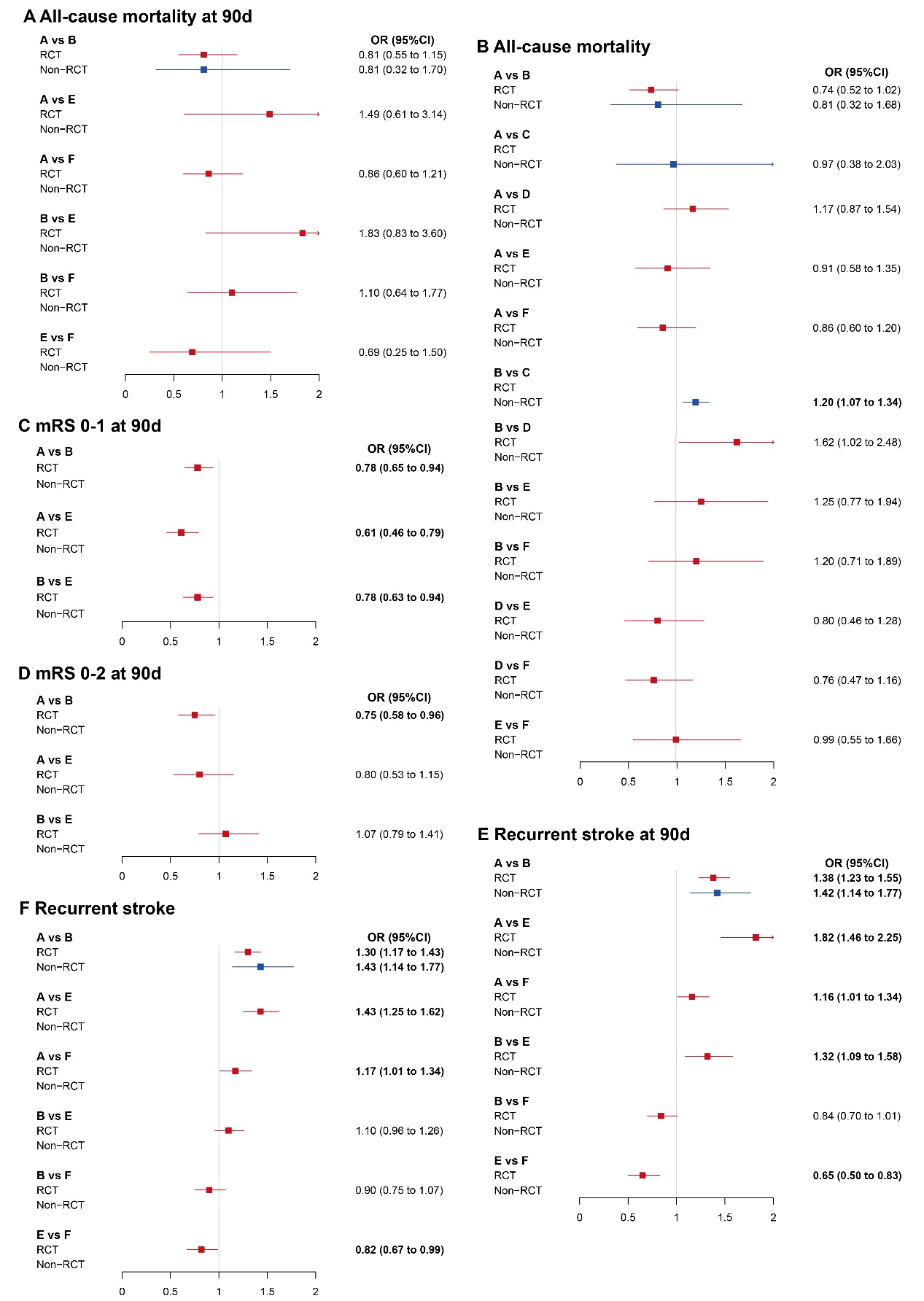
**

**
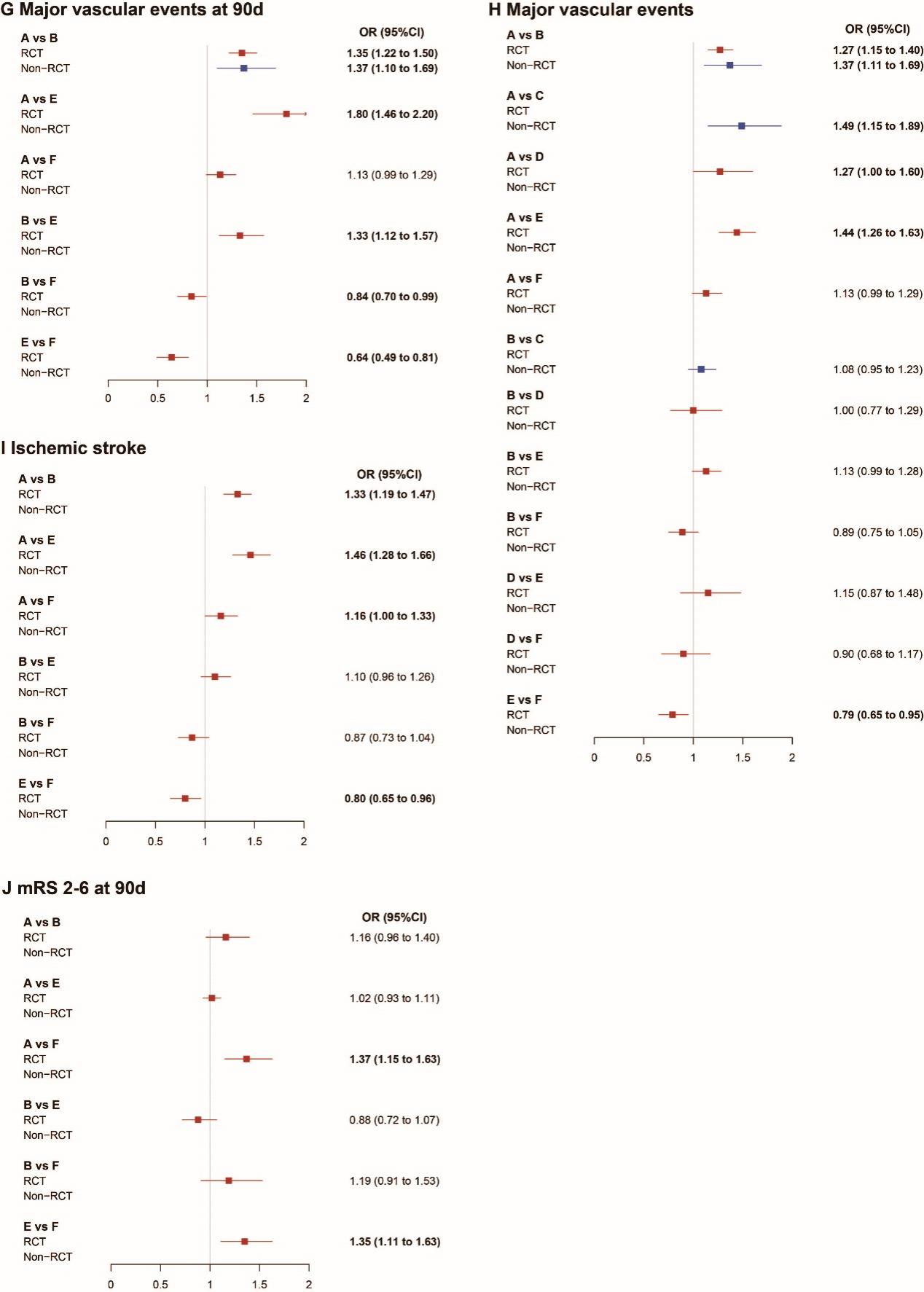
**

**Supplemental Figure 5.** Forest plots for the network meta-analysis of secondary efficacy outcomes. CI, credible interval; A: Aspirin; B: Clopidogrel+Aspirin; C: Cilostazol+Aspirin; D: Dipyridamole+Aspirin; E: Ticagrelor+Aspirin; F: Ticagrelor. mRS, modified rankin scale; OR, odds ratio; RCT, randomized controlled trial.


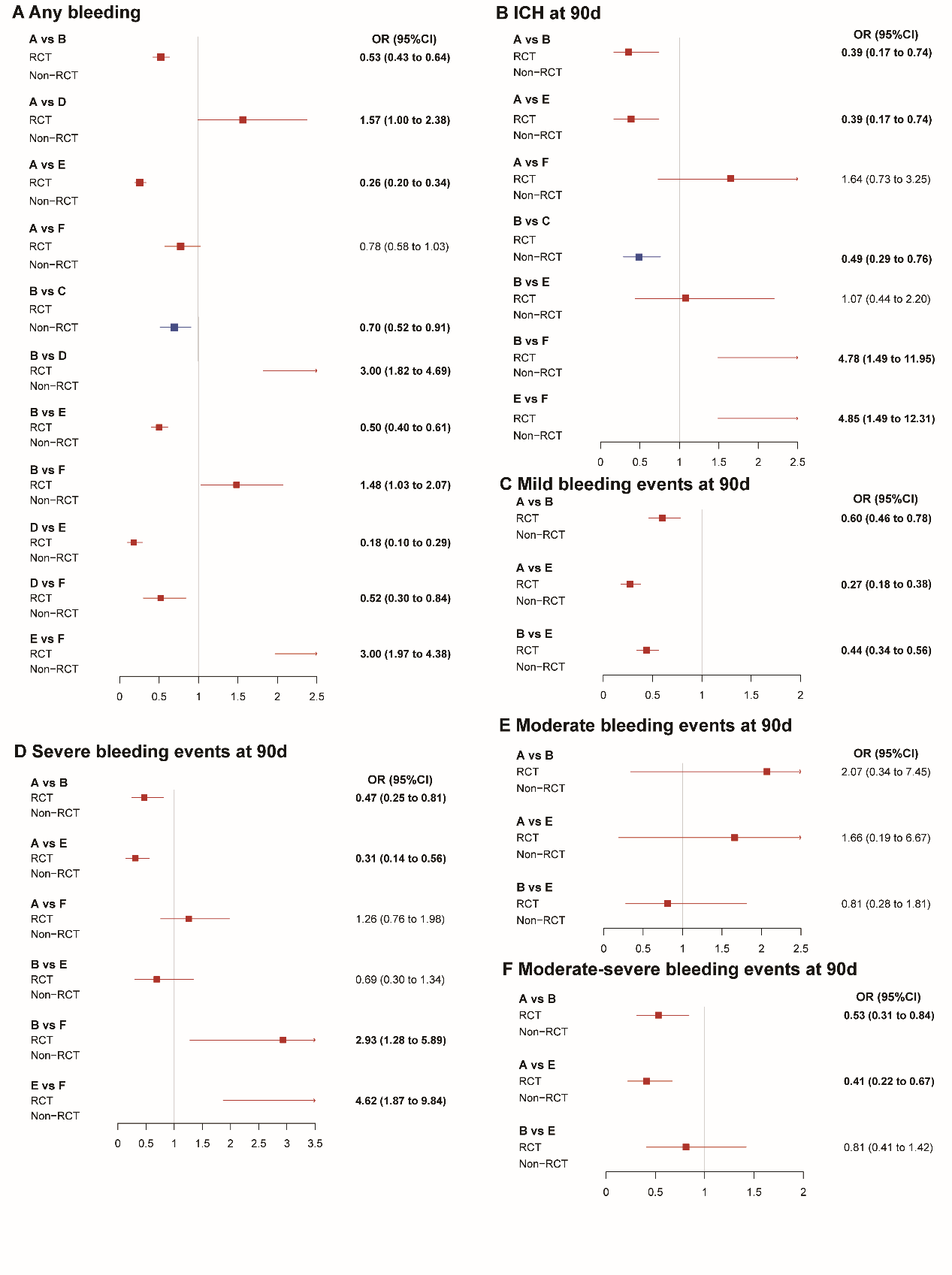
**Supplemental Figure 6.** Forest plots for the network meta-analysis of secondary safty outcomes. CI, credible interval; A: Aspirin; B: Clopidogrel+Aspirin; C: Cilostazol+Aspirin; D: Dipyridamole+Aspirin; E: Ticagrelor+Aspirin; F: Ticagrelor. mRS, modified rankin scale; OR, odds ratio; RCT, randomized controlled trial.


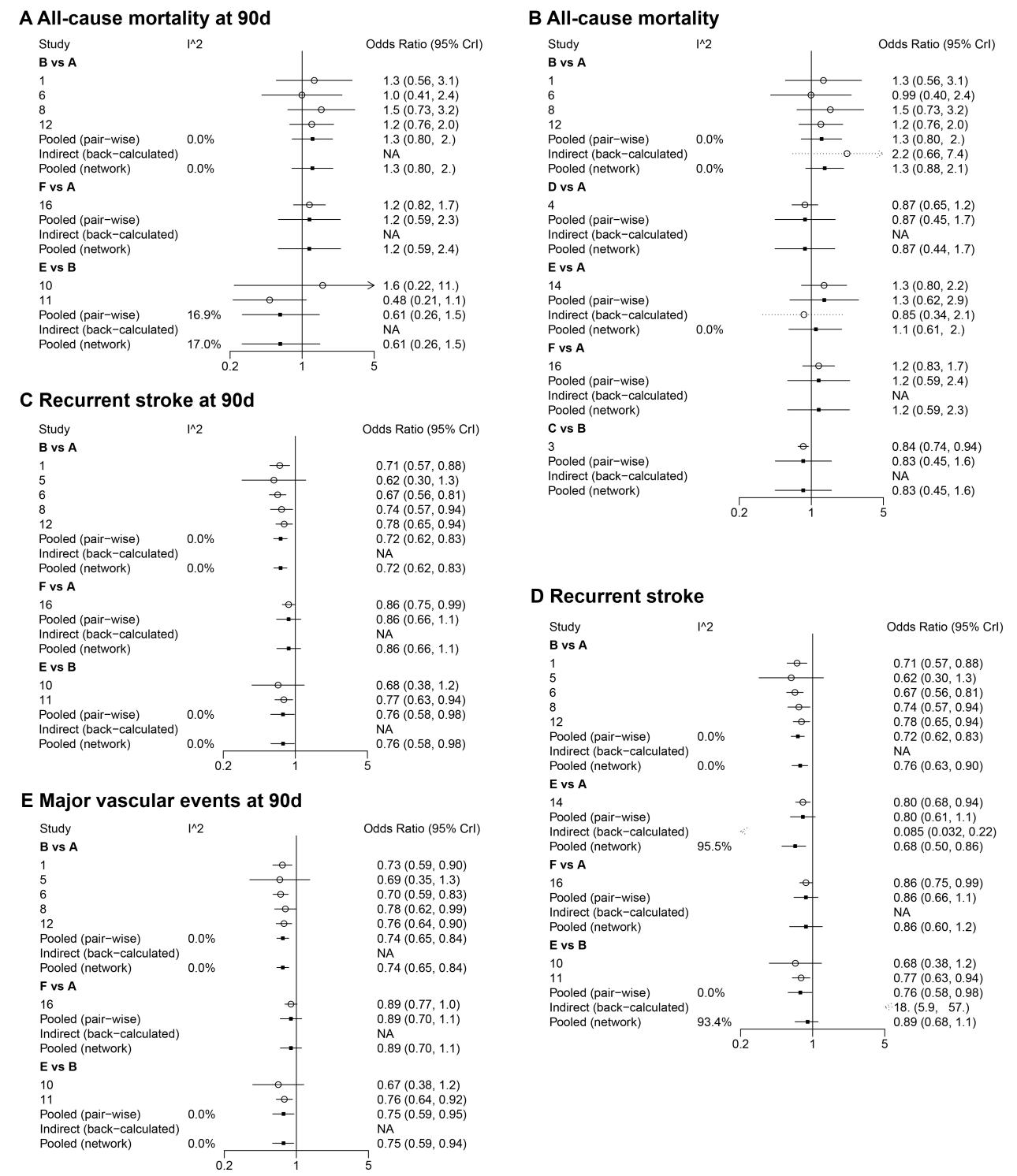


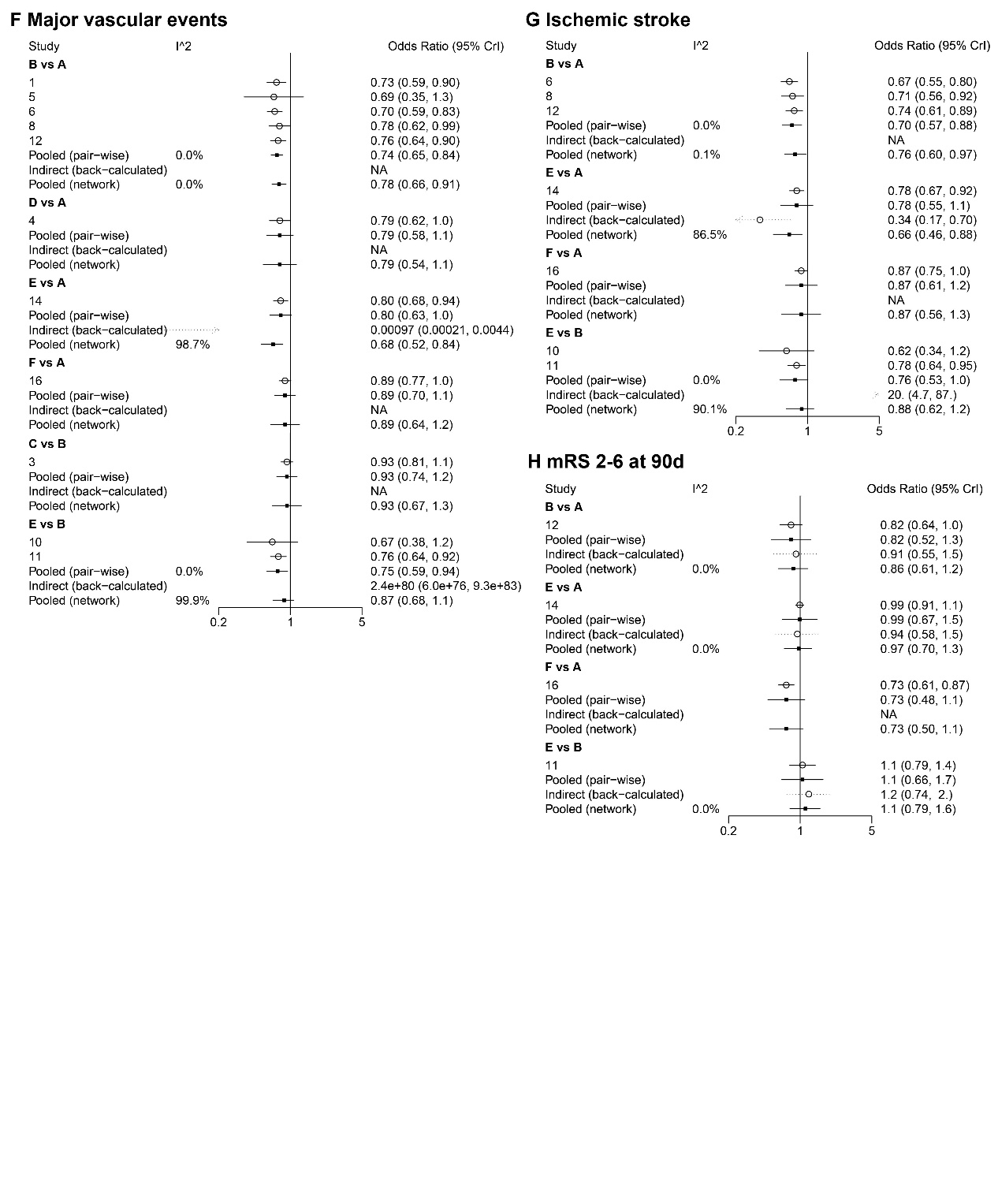


**Supplemental Figure 7.** Heterogeneity of results for efficacy outcomes. A: Aspirin; B: Clopidogrel+Aspirin; C: Cilostazol+Aspirin; D: Dipyridamole+Aspirin; E: Ticagrelor+Aspirin; F: Ticagrelor. mRS, modified rankin scale.


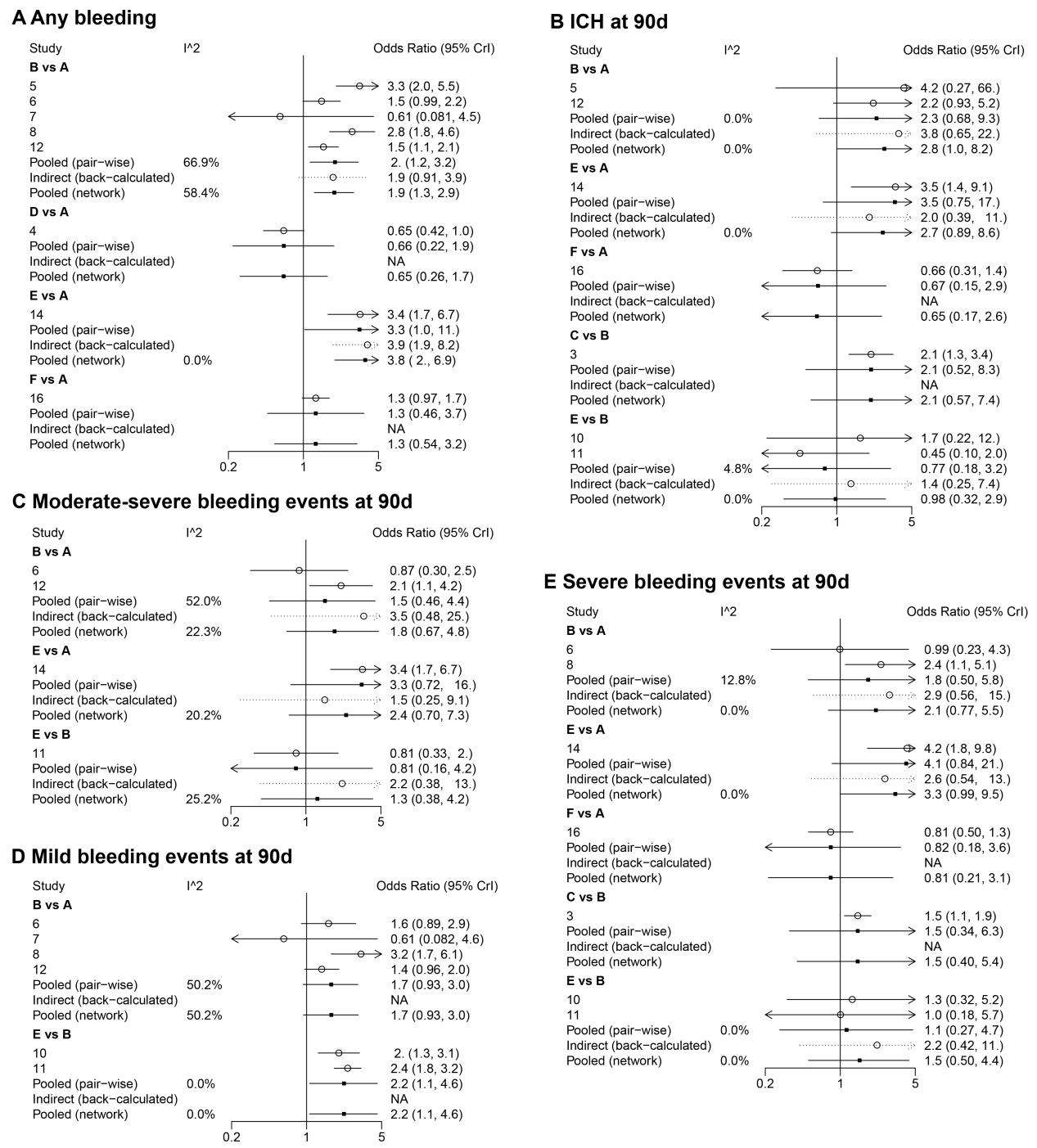


**Supplemental Figure 8.** Heterogeneity of results for safety outcomes. A: Aspirin; B: Clopidogrel+Aspirin; C: Cilostazol+Aspirin; D: Dipyridamole+Aspirin; E: Ticagrelor+Aspirin; F: Ticagrelor. ICH, intracerebral hemorrhage.


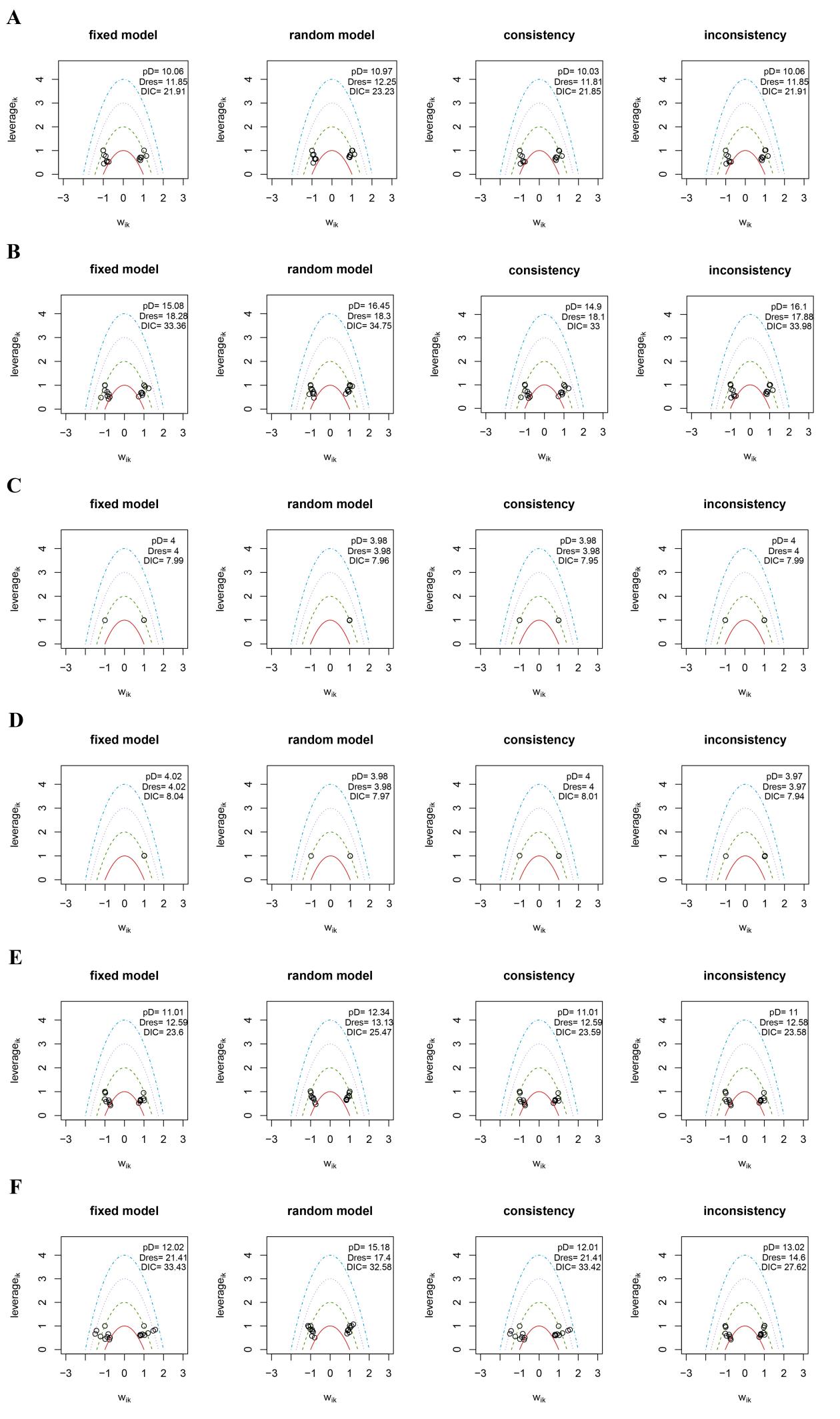


**
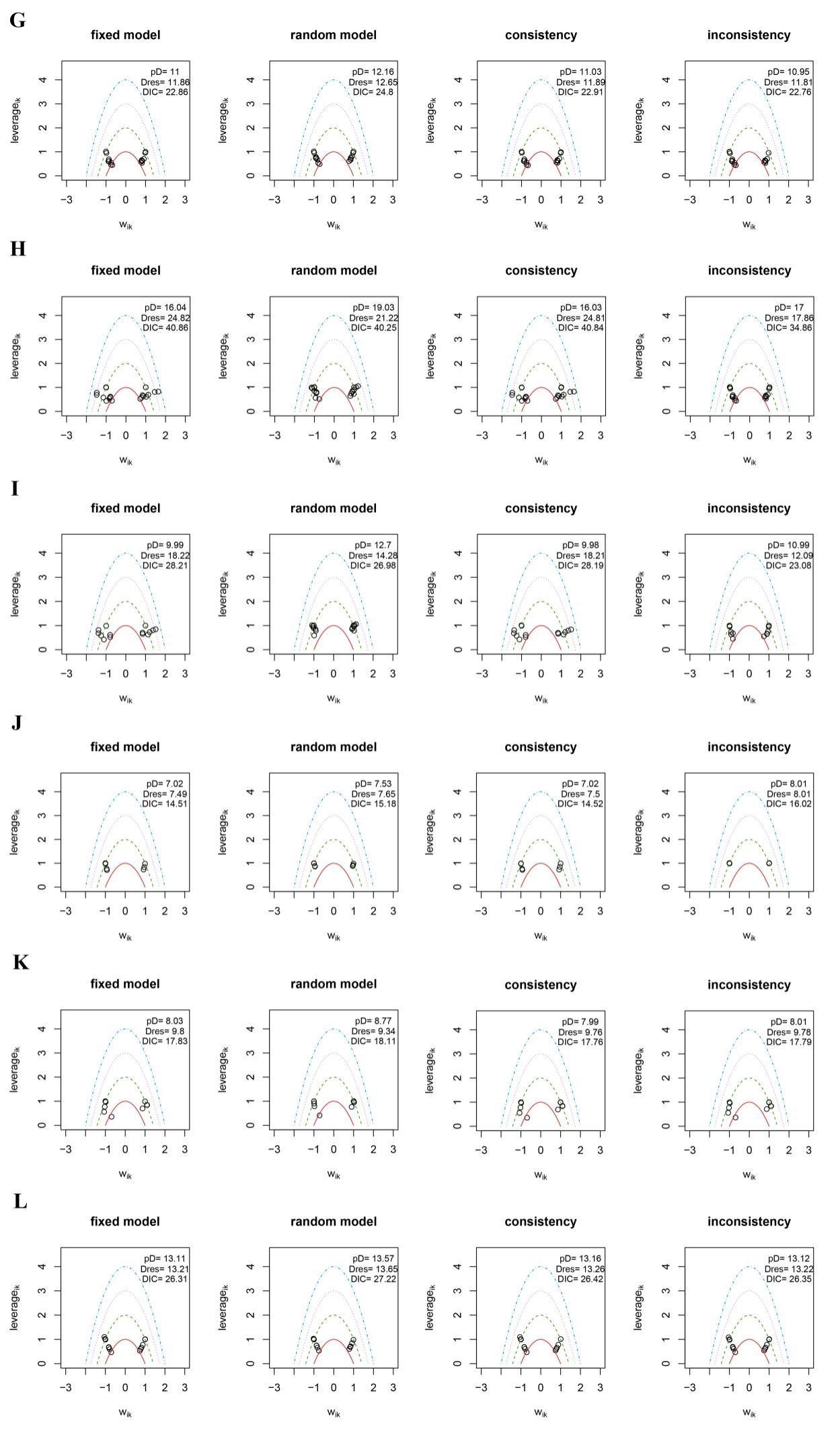
**

**Supplemental Figure 9.** Leverage plot of efficacy outcomes. (A) All-cause mortality at 90d; (B) All-cause mortality; (C) mRS 0-1 at 90d; (D) mRS 0-2 at 90d; (E) Recurrent stroke at 90d; (F) Recurrent stroke; (G) Major vascular events at 90d; (H) Major vascular events; (I) Ischemic stroke; (J) mRS 2-6 at 90d. DIC, deviance information criterion; mRS, modified rankin scale.


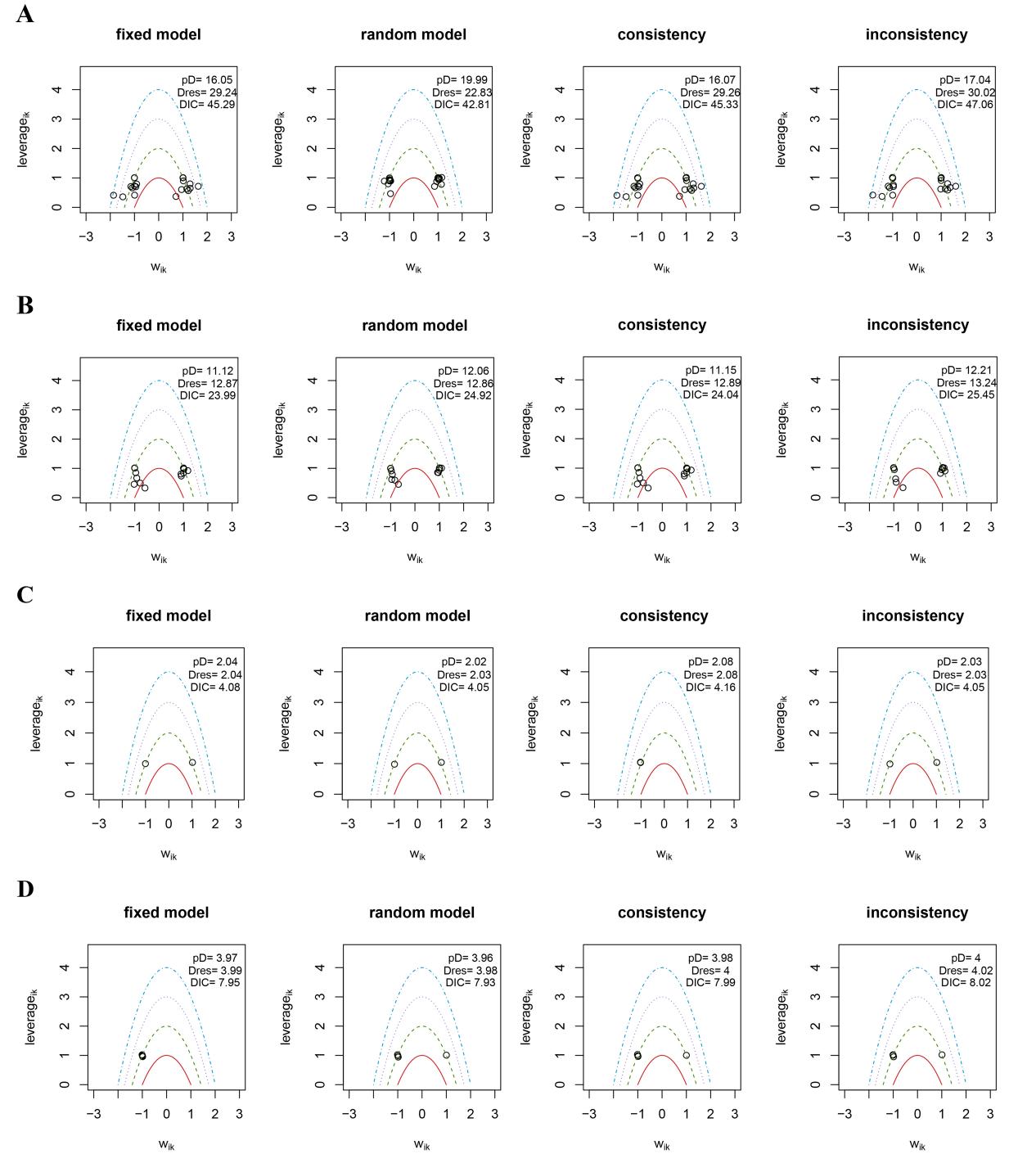


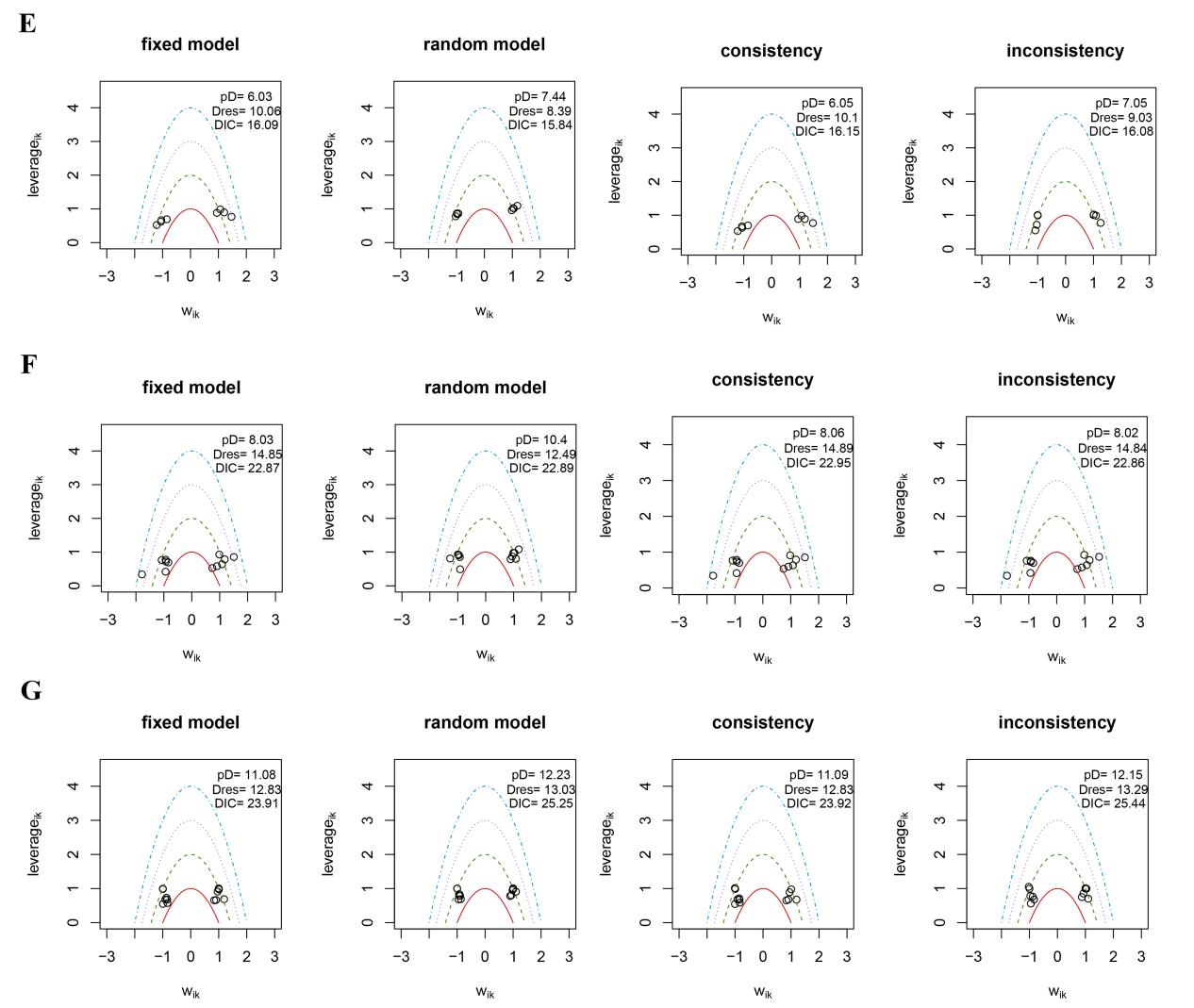


**Supplemental Figure 10.** Leverage plot of safety outcomes. (A) Any bleeding; (B) ICH at 90d; (C) Mild bleeding events at 90d; (D) Severe bleeding events at 90d; (E) Moderate bleeding events at 90d; (F) Moderate-severe bleeding events at 90d. DIC, deviance information criterion; ICH, intracerebral hemorrhage.


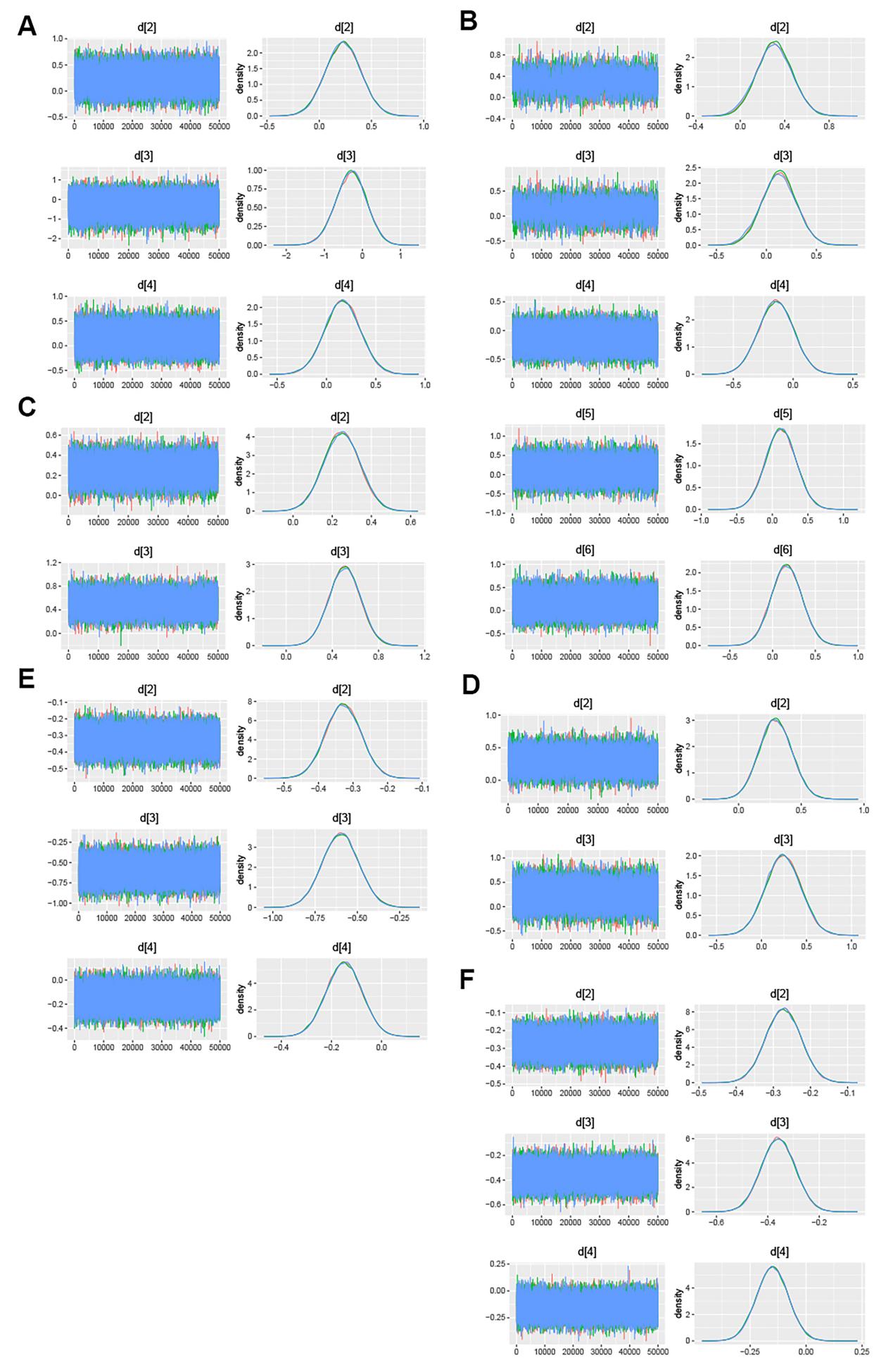


**
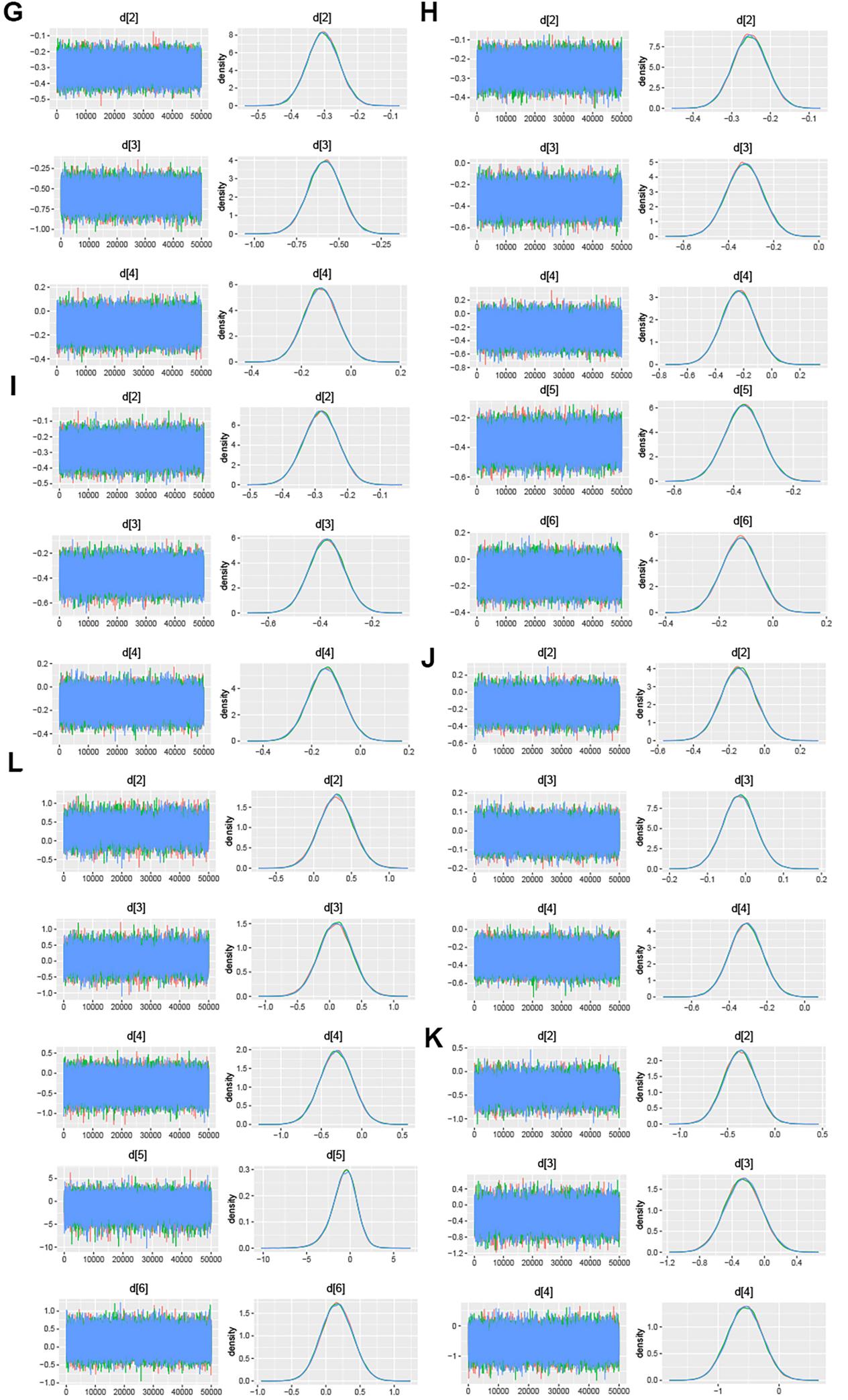
**

**Supplemental Figure 11** The trace and density plots of efficacy outcomes. (A) All-cause mortality at 90d; (B) All-cause mortality; (C) mRS 0-1 at 90d; (D) mRS 0-2 at 90d; (E) Recurrent stroke at 90d; (F) Recurrent stroke; (G) Major vascular events at 90d; (H) Major vascular events; (I) Ischemic stroke; (J) mRS 2-6 at 90d. mRS, modified rankin scale.


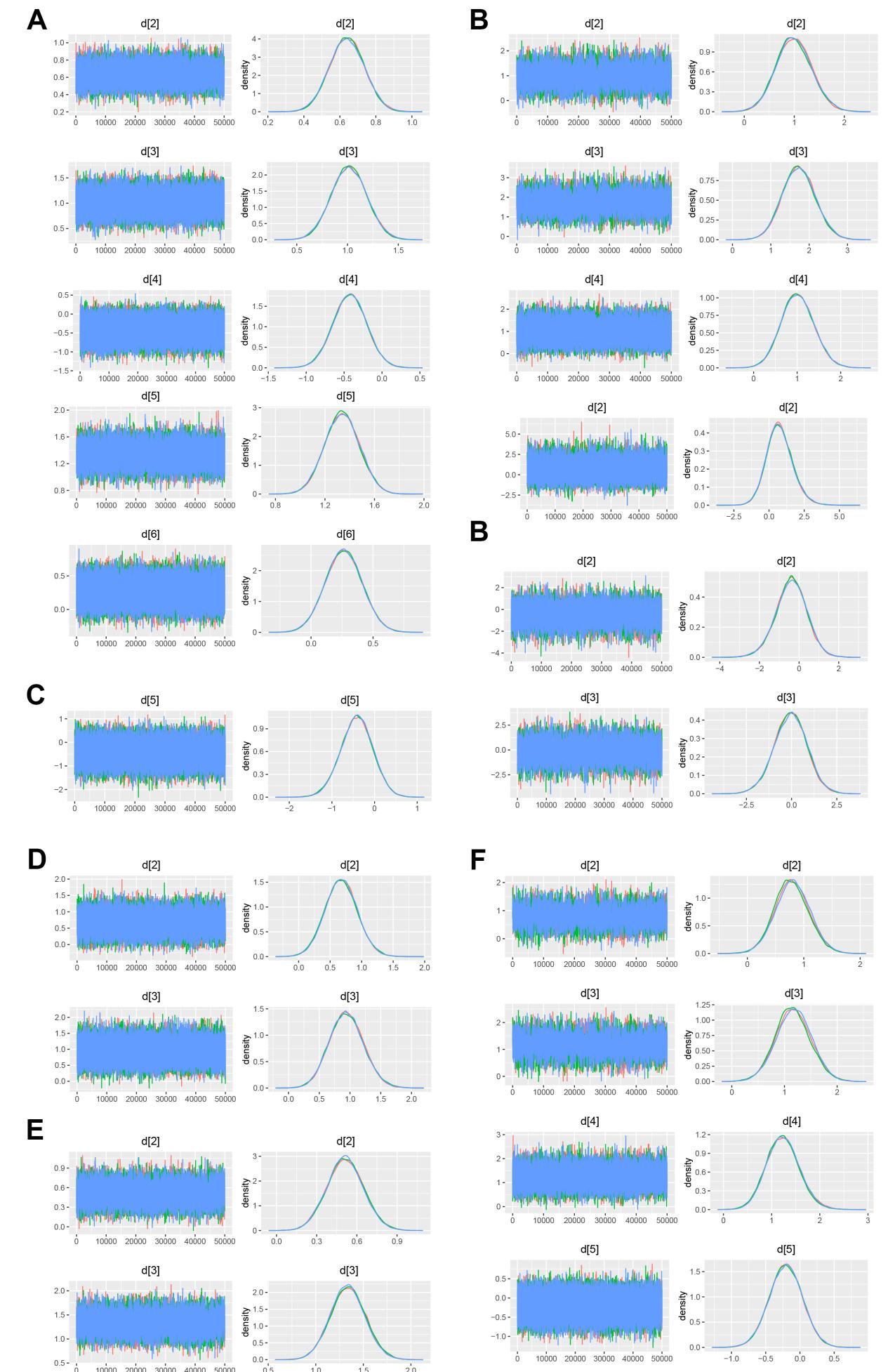


**Supplemental Figure 12.** The trace and density plots of safety outcomes. (A) Any bleeding; (B) ICH at 90d; (C) Mild bleeding events at 90d; (D) Severe bleeding events at 90d; (E) Moderate bleeding events at 90d; (F) Moderate-severe bleeding events at 90d. ICH, intracerebral hemorrhage.


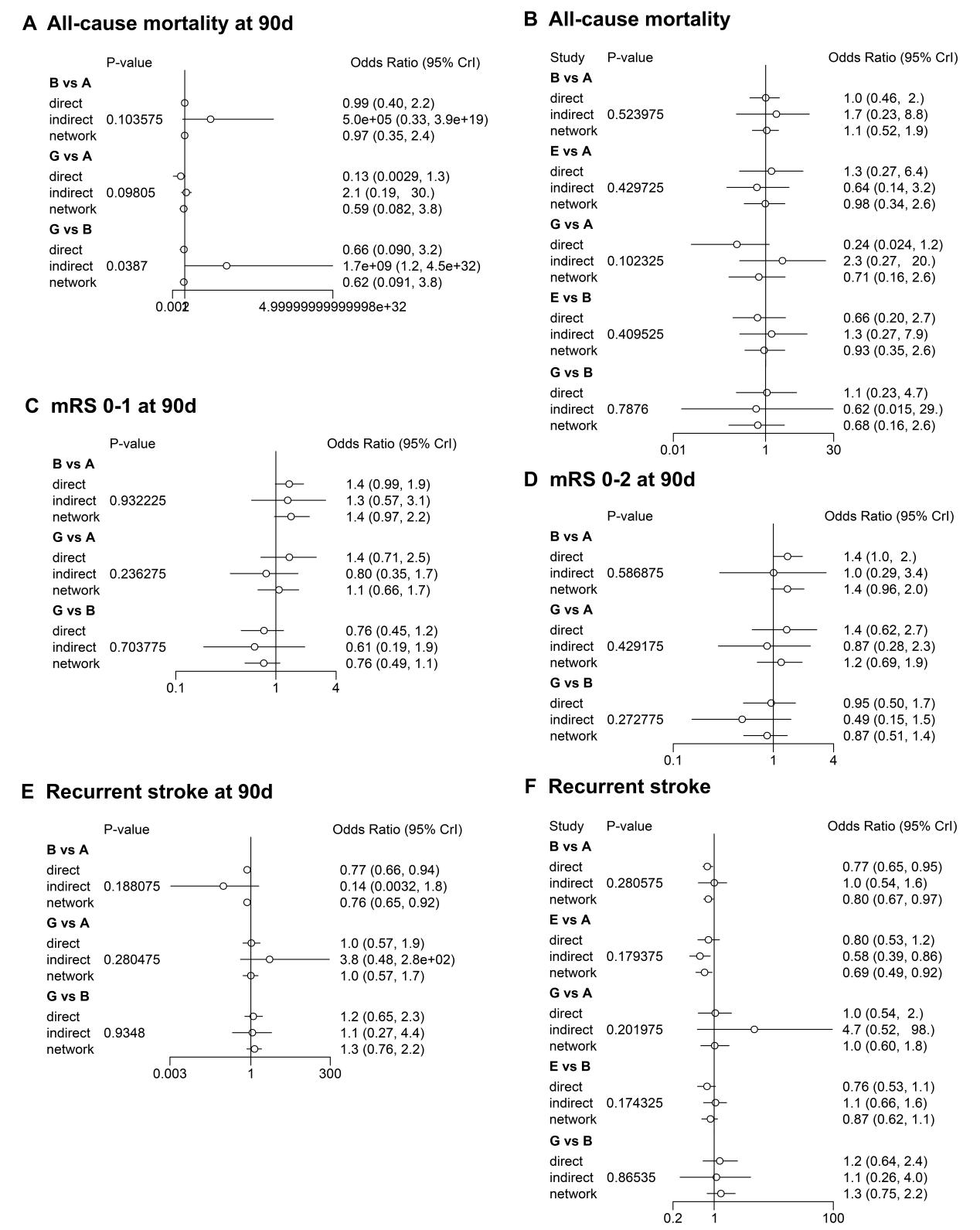


**
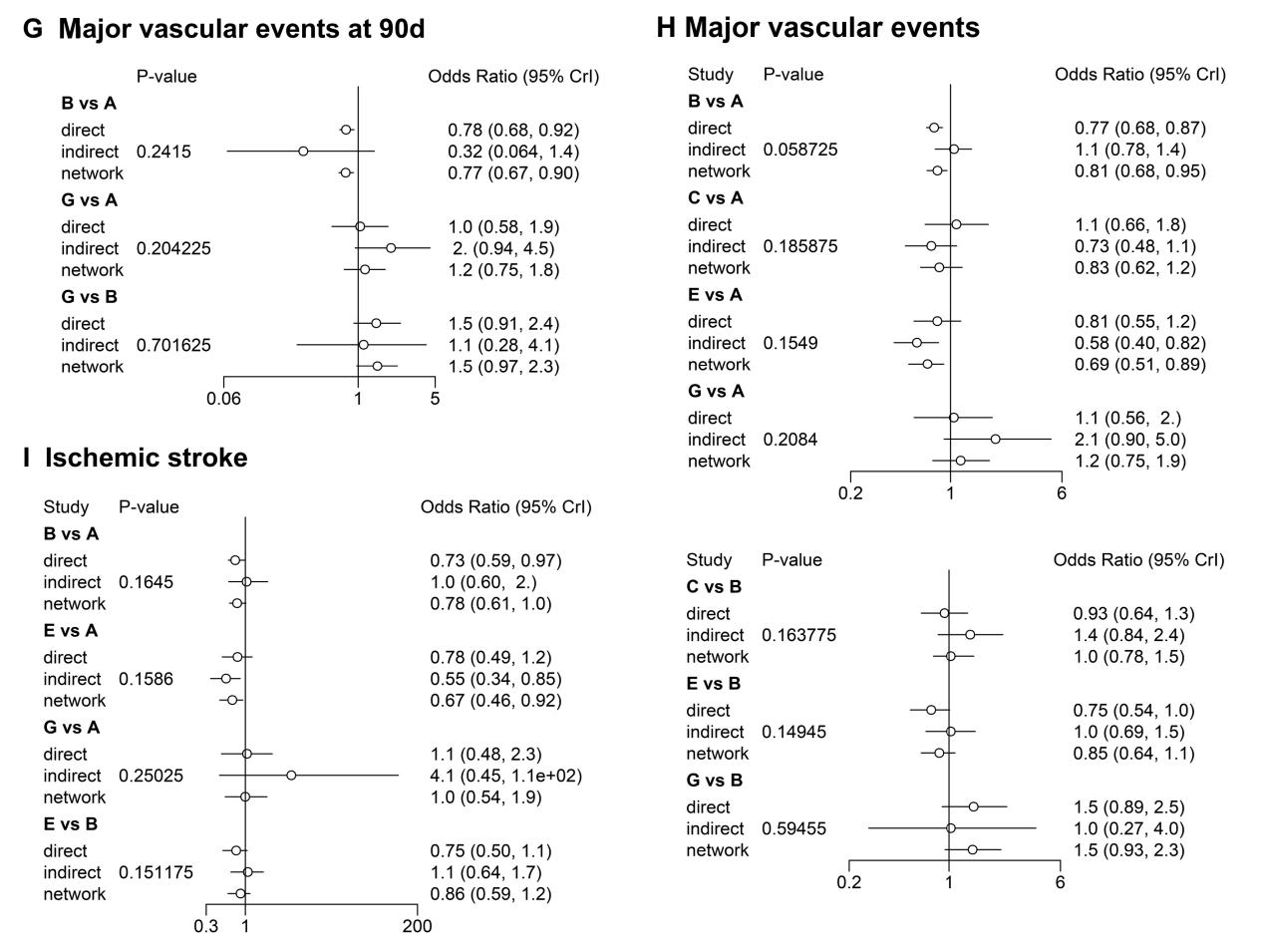
**

**Supplemental Figure 13.** Nodes-splitting analysis of efficacy outcomes. P > 0.05 indicates that there is no significant inconsistency which means the direct, indirect and network comparison is consistent. A: Aspirin; B: Clopidogrel+Aspirin; C: Cilostazol+Aspirin; D: Dipyridamole+Aspirin; E: Ticagrelor+Aspirin; F: Ticagrelor. mRS, modified rankin scale; TIA, transient ischemic attack.


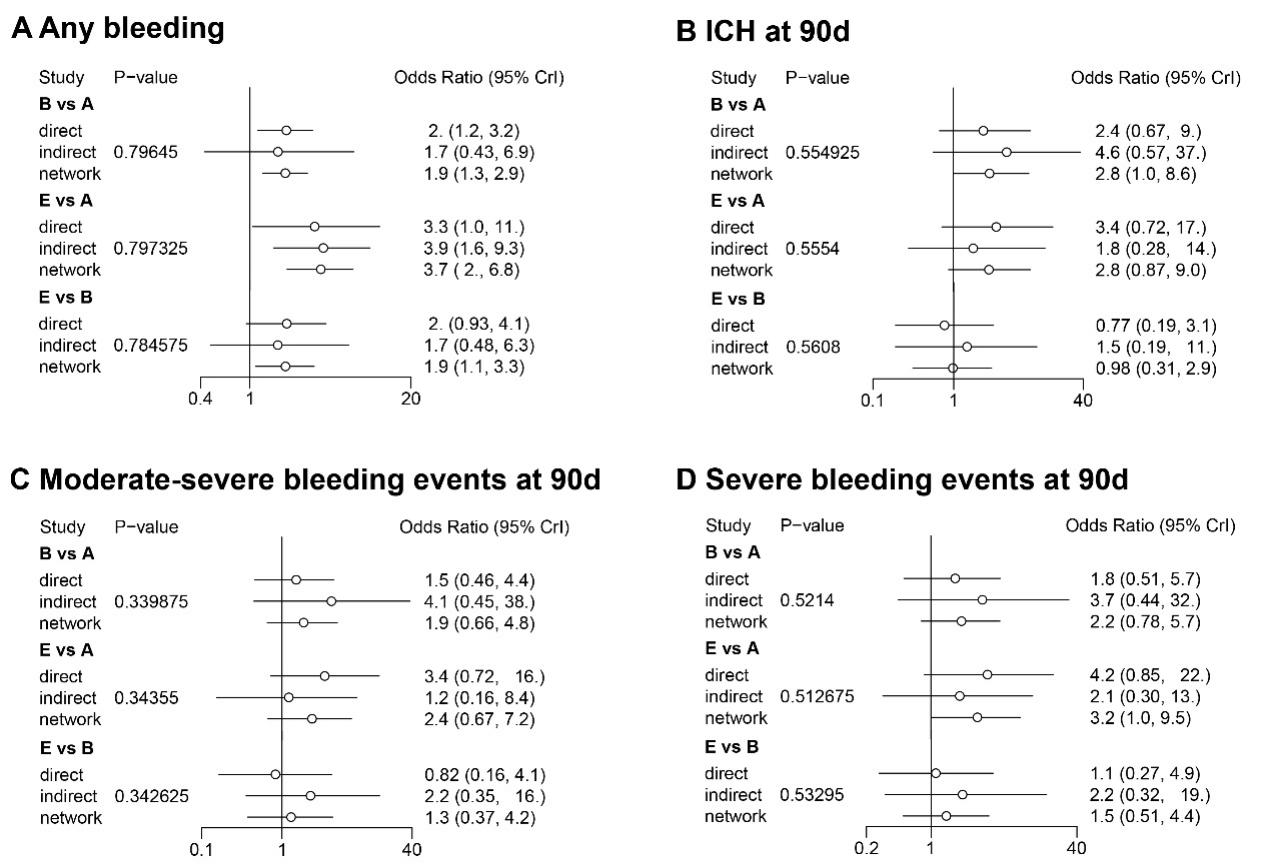


**Supplemental Figure 14.** Nodes-splitting analysis of safety outcomes. P > 0.05 indicates that there is no significant inconsistency which means the direct, indirect and network comparison is consistent. A: Aspirin; B: Clopidogrel+Aspirin; C: Cilostazol+Aspirin; D: Dipyridamole+Aspirin; E: Ticagrelor+Aspirin; F: Ticagrelor. ICH, intracerebral hemorrhage.


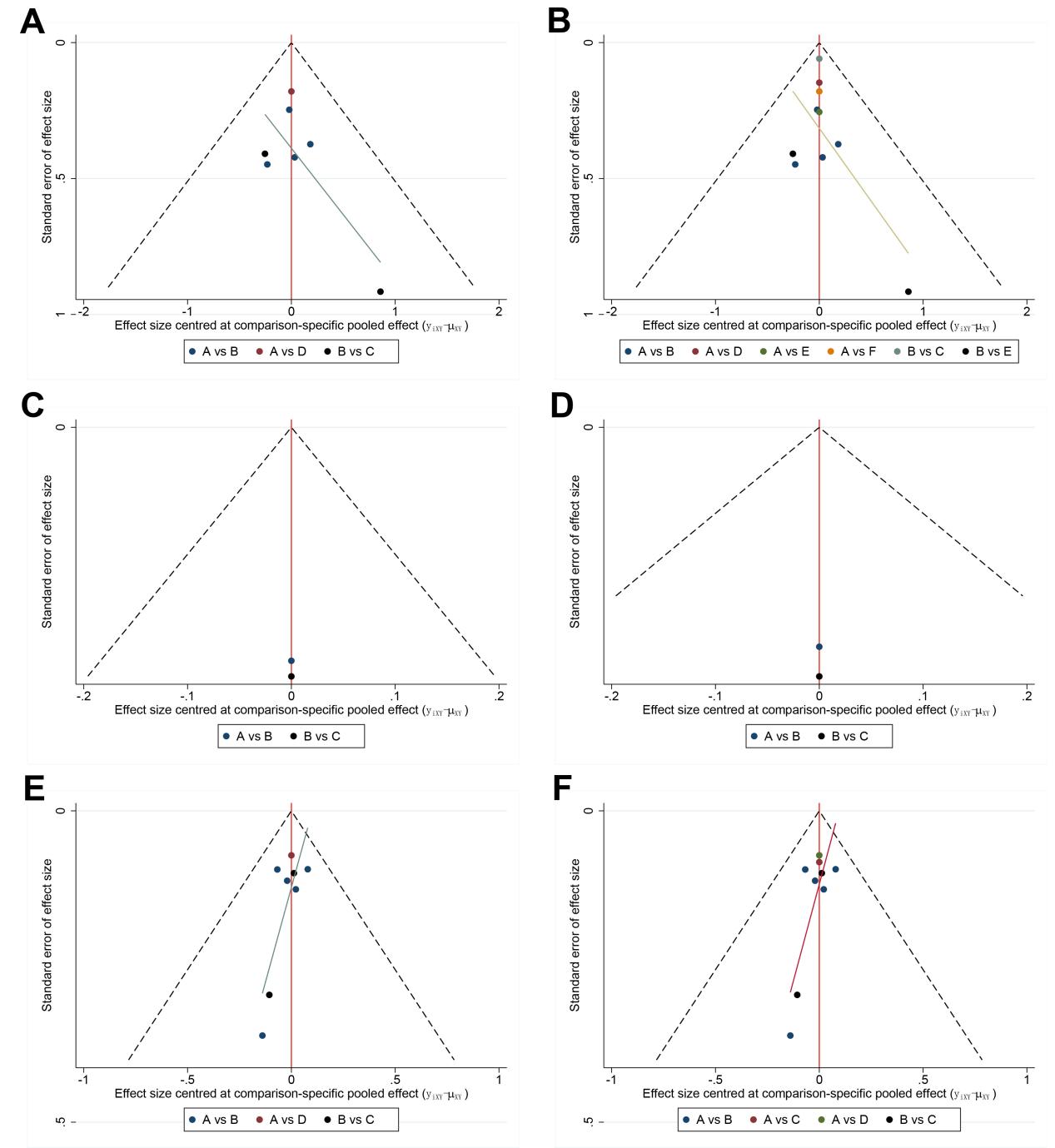


**
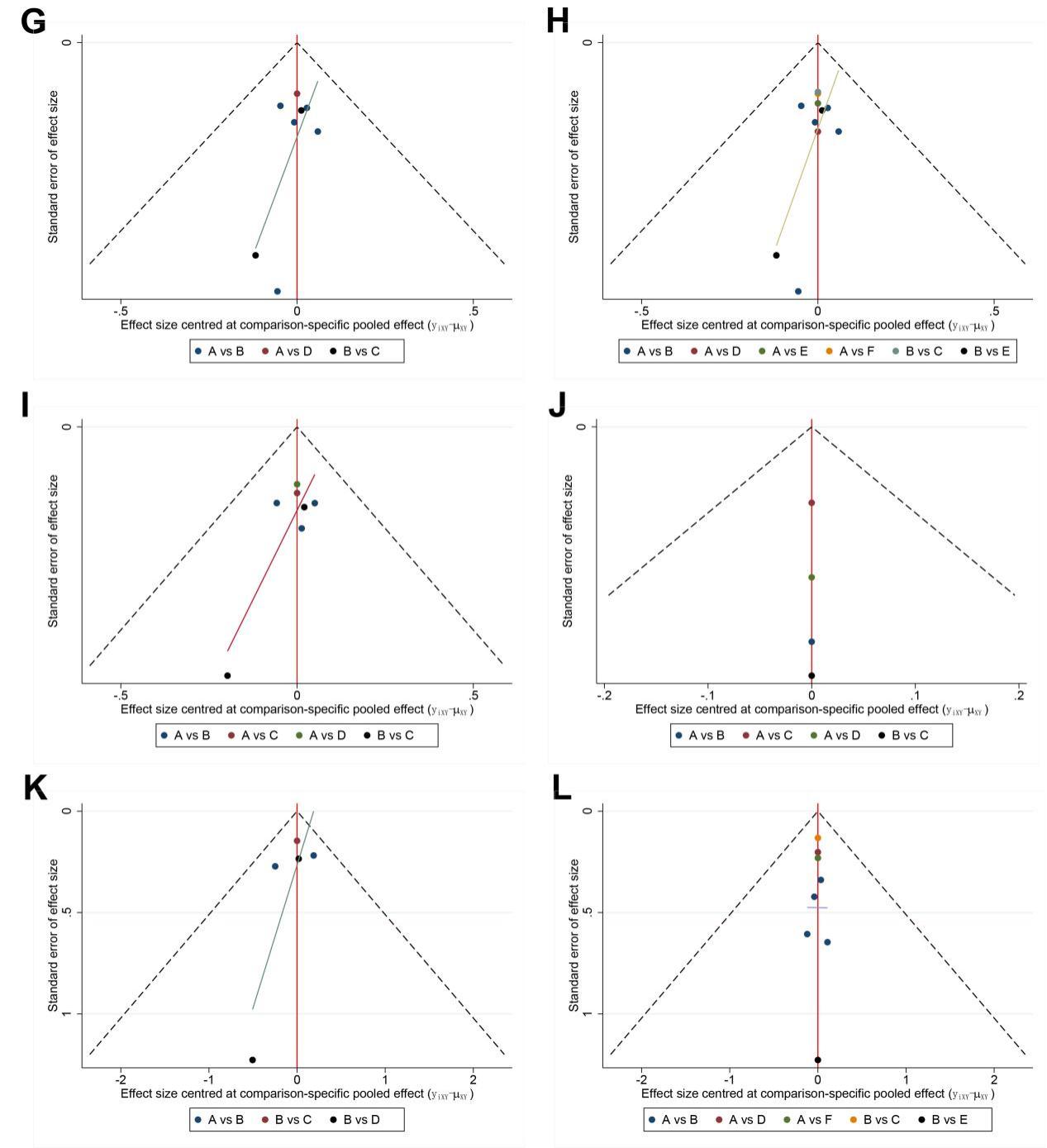
**

**Supplemental Figure 15.** Publication Bias Assessment of efficacy outcomes. (A) All-cause mortality at 90d; (B) All-cause mortality; (C) mRS 0-1 at 90d; (D) mRS 0-2 at 90d; (E) Recurrent stroke at 90d; (F) Recurrent stroke; (G) Major vascular events at 90d; (H) Major vascular events; (I) Ischemic stroke; (J) mRS 2-6 at 90d. A: Aspirin; B: Clopidogrel+Aspirin; C: Cilostazol+Aspirin; D: Dipyridamole+Aspirin; E: Ticagrelor+Aspirin; F: Ticagrelor. mRS, modified rankin scale.


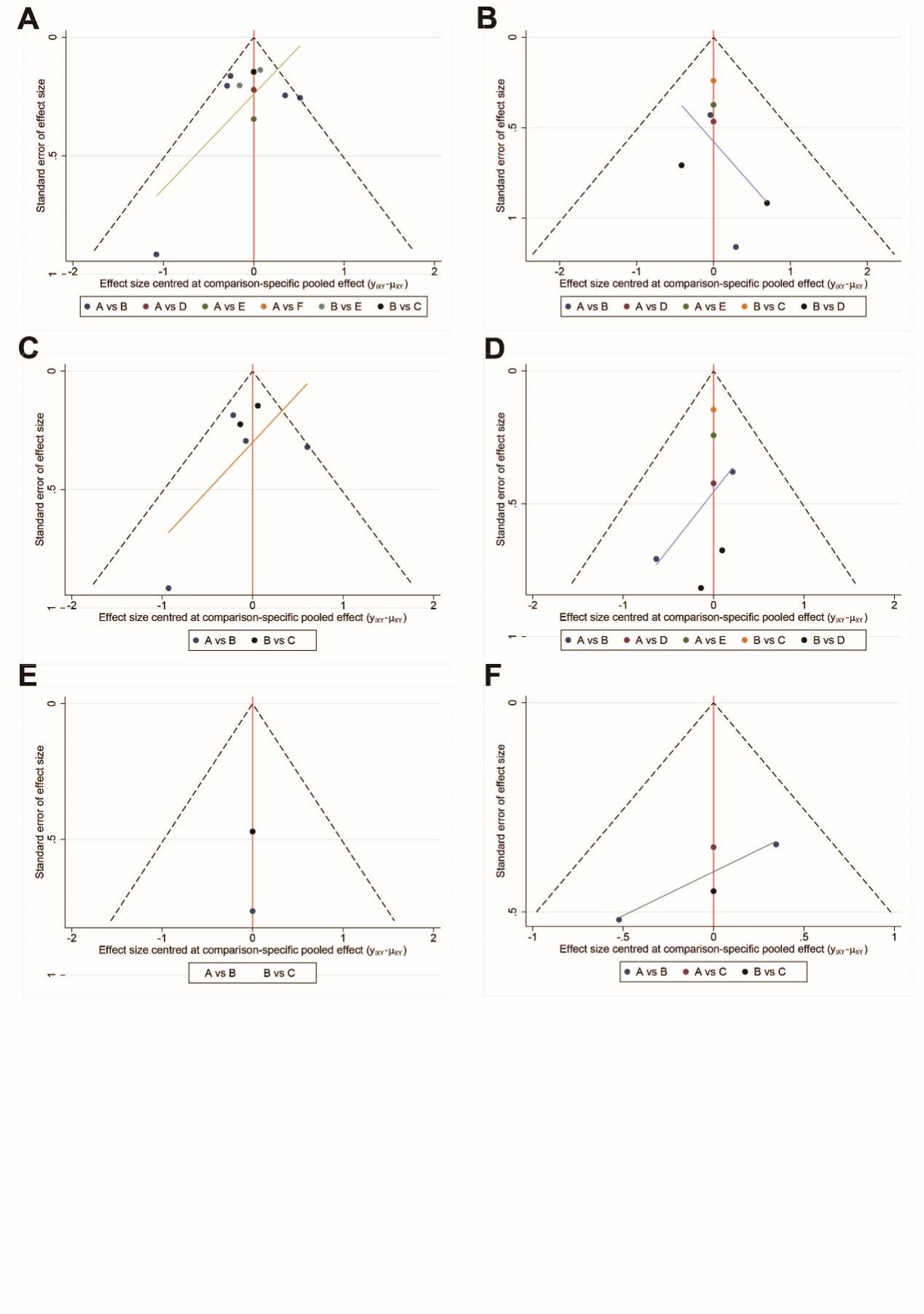


**Supplemental Figure 16.** Publication Bias Assessment of safety outcomes. (A) Any bleeding; (B) ICH at 90d; (C) Mild bleeding events at 90d; (D) Severe bleeding events at 90d; (E) Moderate bleeding events at 90d; (F) Moderate-severe bleeding events at 90d. A: Aspirin; B: Clopidogrel+Aspirin; C: Cilostazol+Aspirin; D: Dipyridamole+Aspirin; E: Ticagrelor+Aspirin; F: Ticagrelor. ICH, intracerebral hemorrhage.

**Supplemental Table 2** Characteristics of the included trials

| Study | Country | Study design | Time period | T/C | Sample size (M/F) | Age | Intervention and control protocol | | Stroke type | Severity of stroke and TIA | Treatment onset | Dosages | Duration of treatment | Follow up | Analysis |
| --- | --- | --- | --- | --- | --- | --- | --- | --- | --- | --- | --- | --- | --- | --- | --- |
|  |  |  |  |  |  |  | T | C |  |  |  |  |  |  |  |
| Gao et al. 2023 | China | RCT | 2018-2022 | 3050/3050 | 6100 3915/2185 | T: 65 C: 65 | Clop+Asp | Asp | Minor stroke or TIA | NIHSS ≤ 3 ABCD2 ≥ 4 | ≤24 h | **Loading:** aspirin 100-300 mg or aspirin 100-300 mg plus clopidogrel 300 mg. **Maintenance:** aspirin and clopidogrel were 100 mg daily and 75 mg daily. | 90 days | 270 days | ITT |
| Halkes et al. 2006 | Austria | RCT | 1997-2005 | 1363/1376 | 2739 1789/950 | T: 63 C: 63 | Dipy +Asp | Asp | Minor stroke or TIA | NR | NR | Aspirin (30–325 mg daily) with or without dipyridamole (200 mg twice daily) | 180 days | ≤5 years | ITT |
| He et al. 2014 | China | RCT | 2010-2012 | 321/ 326 | 647 368/279 | T: 62.92  C: 61.52 | Clop+Asp | Asp | Minor stroke or TIA | NIHSS ≤ 7 ABCD2 ≥ 4 | ≤72 h | **Loading:** aspirin 300 mg or aspirin 300 mg plus clopidogrel 300 mg. **Maintenance:** aspirin and clopidogrel were 100 mg daily and 75 mg daily. | 14 days | 14 days | ITT |
| Huang et al. 2023 | China | Prospective study | 2008-2018 | 578/2825 | 6838.4  2133/1270 | T: 68.76 C: 68.52 | Cilo＋Asp | Clop＋Asp | Minor stroke or TIA | NIHSS ≤ 4 | NR | In the first 30 days, both groups were treated with double antiplatelet therapy, followed by double antiplatelet therapy or single antiplatelet therapy. | 30 days | T: 1.67  years  C: 2.08  years | IPTW |
| Johnston et al.2016 | USA | RCT | 2014-2015 | 6589/6610 | 13199  7716/5483 | T: 65.8  C: 65.9 | Tica | Asp | Minor stroke or TIA | NIHSS ≤ 5 ABCD2 ≥ 4 | ≤24 h | **Loading:** ticagrelor 180 mg or aspirin 300 mg.  **Maintenance:** ticagrelor 90 mg twice daily or aspirin 100 mg daily | 90 days | 90 days | ITT |
| Johnston et al. 2018 | USA | RCT | 2010-2017 | 2432/2449 | 4881 2686/2195 | T: 65 C: 65 | Clop+Asp | Asp | Minor stroke or TIA | NIHSS ≤ 3 ABCD2 ≥ 4 | ≤12 h | **Loading:** aspirin 75-325 mg or aspirin 75-325 mg plus clopidogrel 600 mg. **Maintenance:** aspirin and clopidogrel were 81 mg daily and 75 mg daily. | 90 days | 90 days | ITT |
| Johnston et al. 2020 | Sweden | RCT | 2018-2019 | 5523/5493 | 11016 6737/4279 | T: 65.2 C: 65.1 | Tica+Asp | Asp | Minor stroke or TIA | NIHSS ≤5 | ≤24 h | **Loading:** aspirin 300-325 mg or aspirin 300-325 mg plus ticagrelor 180 mg. **Maintenance:** aspirin and clopidogrel were 75-100 mg daily and 90 mg bid. | 30 days | 30 days | ITT |
| Kennedy et al. 2007 | UK | RCT | 2003-2006 | 201/ 195 | 396 211/185 | T: 69.8  C: 68.9 | Clop+Asp | Asp | Minor stroke or TIA | NIHSS≤3 | ≤24 h | **Loading:** aspirin 162 mg or aspirin 162 mg plus clopidogrel 300 mg. **Maintenance:** aspirin and clopidogrel were 81 mg dailyand 75 mg daily. | 90 days | 90 days | ITT |
| Kim et al. 2019 | Korea | Prospective study | 2008-2016 | 1604/1604 | 3208 2006/1202 | T: 64.8  C: 64.9 | Clop+Asp | Asp | Minor stroke or TIA | NIHSS ≤ 3 ABCD2 ≥ 4 | ≤24 h | NR | NR | 90 days | PSM |
| Wang et al. 2013 | China | RCT | 2009-2012 | 2584/2586 | 5170 3420/1750 | T: 63 C: 62 | Clop+Asp | Asp | Minor stroke or TIA | NIHSS ≤ 3 ABCD2 ≥ 4 | ≤24 h | **Loading:** aspirin 75 mg or aspirin 75 mg plus clopidogrel 300 mg. **Maintenance:** aspirin and clopidogrel were 75 mg daily and 75 mg daily. | 21 days | 90 days | ITT |
| Wang et al. 2019 | China | RCT | 2015-2017 | 336/339 | 675  494/181 | T:61.1  C: 60.5 | Tica+Asp | Clop+Asp | Minor stroke or TIA | NIHSS ≤ 3 ABCD2 ≥ 4 | ≤24 h | **Loading:** aspirin 100-300 mg on day 1, followed by 100 mg once daily until day 21 **Maintenance:** aspirin plus ticagrelor (180 mg on day 1, followed by 90 mg twice daily) or aspirin plus clopidogrel (300 mg on day 1, followed by 75 mg once daily). | 90 days | 180 days | ITT |
| Wang et al. 2021 | China | RCT | 2019-2021 | 3205/3207 | 6412 4242/2170 | T:65.0 C: 64.6 | Tica–Asp | Clop+Asp | Minor stroke or TIA | NIHSS ≤ 3 ABCD2 ≥ 4 | ≤24 h | **Loading:** aspirin 100-300 mg on day 1, followed by 100 mg once daily until day 21 **Maintenance:** aspirin plus ticagrelor (180 mg on day 1, followed by 90 mg twice daily) or aspirin plus clopidogrel (300 mg on day 1, followed by 75 mg once daily). | 90 days | 180 days | ITT |

Asp: Aspirin; Clop: Clopidogrel; Cilo: Cilostazol; Dipy: Dipyridamole; ITT: Intention-to-treat; M/F: Male/female; mRS, modified Rankin Scale; NIHSS: National Institute of Health stroke scale; NR: No reported; PSM: Propensity Score Matching; T/C: treatment group/control group; Tica: Ticagrelor; RCT, randomized controlled trial; UK: United Kingdom; USA: The United States of America.

**Supplemental Table 3:** All-cause mortality outcomes in each study

| First author and year | All-cause mortality | Comments |
| --- | --- | --- |
| Gao et al. 2023 | 90 days | Cardiovascular death |
| Halkeset al. 2006 | 3-5 years |  |
| He et al. 2014 | 90 days |  |
| Huang et al. 2023 | 90 days | Mortality: fatal stroke, cardiovascular mortality, all-cause mortality  Fatal stroke: deaths resulting from ischemic or hemorrhagic strokes  Cardiovascular mortality: deaths caused by ischemic heart diseases, cardiac arrhythmia, heart failure, or cerebrovascular diseases |
| Johnston et al.2016 | 90 days |  |
| Johnston et al. 2018 | 90 days |  |
| Johnston et al. 2020 | 90 days | From randomization through 30 days of follow-up |
| Kennedy et al. 2007 | 90 days |  |
| Kim et al. 2019 | 90 days | Vascular death |
| Wang et al. 2013 | 90 days |  |
| Wang et al. 2019 | 90 days |  |
| Wang et al. 2021 | 90 days |  |

**Supplemental Table 4.** Bleeding definitions

| First author and year | Bleeding definitions |
| --- | --- |
| Gao et al. 2023 | Moderate to severe bleeding events: GUSTO criteria |
| Halkeset al. 2006 | Intracranial haemorrhages: subdural and epidural haematomas.  Major bleeding: all intracranial bleeding, any fatal bleeding, any bleeding requiring hospital admission. |
| He et al. 2014 | Not reported |
| Huang et al. 2023 | Composite major bleeding events: ICH and gastrointestinal bleeding |
| Johnston et al.2016 | Any bleeding: Major or minor bleeding, PLATO criteria  Severe bleeding events: Major bleeding, PLATO criteria |
| Johnston et al. 2018 | Major hemorrhage: symptomatic intracranial hemorrhage, intraocular bleeding causing vision loss, transfusion of 2 or more units of red cells or an equivalent amount of whole blood, hospitalization or prolongation of an existing hospitalization, or death due to hemorrhage. |
| Johnston et al. 2020 | Hemorrhagic stroke: symptomatic intraparenchymal, intraventricular, or subarachnoid hemorrhage  Composite events: first intracranial hemorrhage or fatal bleeding event |
| Kennedy et al. 2007 | Haemorrhagic events: WHO criteria, intracranial and extracranial groups  Severe extracranial hemorrhage: life threatening, resulting in haemodynamic compromise or hypovolaemic shock, requiring inotropic support or other means to maintain cardiac output, requiring blood transfusion of more than 2 units of packed red blood cells, or associated with a fall in haemoglobin greater than or equal to 5 g/L  Moderate extracranial hemorrhage: requiring a transfusion of 2 units of packed red blood cells or less, not severe as defined above, or associated with a fall in haemoglobin of less than 5 g/L  Mild extracranial hemorrhage: bleeding not requiring transfusion, not causing haemodynamic compromise, usually including haematoma, subcutaneous bleeding, oozing from puncture sites, and may require modification of drug regimen  And asymptomatic extracranial hemorrhage: bleeding that results in no symptoms |
| Kim et al. 2019 | Not reported |
| Wang et al. 2013 | Hemorrhagic stroke: acute extravasation of blood into the brain parenchyma or subarachnoid space with associated neurologic symptoms. Recurrent stroke was considered to be disabling if the score on the modified Rankin scale was 2 or more  Moderate-to-severe bleeding event: GUSTO criteria  Severe hemorrhage: fatal or intracranial hemorrhage or other hemorrhage causing hemodynamic compromise that required blood or fluid replacement, inotropic support, or surgical intervention  Moderate hemorrhage: bleeding that required transfusion of blood but did not lead to hemodynamic compromise requiring intervention |
| Wang et al. 2019 | Major bleeding: PLATO study (fatal or life threatening bleed, major bleed, and other ) |
| Wang et al. 2021 | Severe or moderate bleeding: GUSTO criteria  Mild bleeding: GUSTO criteria |

ECASS II: European Cooperative Acute Stroke Study II; ECASS III: European Cooperative Acute Stroke Study III; GUSTO: Global Utilization of Streptokinase and t-PA for Occluded Coronary Arteries; NIHSS: National Institute of Health stroke scale; sICH: symptomatic intracerebral hemorrhage; sHT: symptomatic hemorrhagic transformation.

**Supplemental Table 5:** Major vascular events definitions

| First author and year | Major vascular events |
| --- | --- |
| Gao et al. 2023 | stroke, myocardial infarction, death from cardiovascular causes |
| Halkeset al. 2006 | All major ischaemic events: non-haemorrhagic death from vascular causes, non-fatal ischaemic stroke, non-fatal myocardial infarction |
| He et al. 2014 | Stroke recurrence: additional neurological deficit and corresponding positive lesions on DWI. |
| Huang et al. 2023 | Composite cardiovascular events: acute ischemic stroke, TIA, AMI |
| Johnston et al.2016 | Primary end point: Stroke, myocardial infarction, or death  Composite of stroke: ischemic or hemorrhagic |
| Johnston et al. 2018 | Ischemic stroke, myocardial infarction, or death from ischemic vascular causes |
| Johnston et al. 2020 | Ischemic stroke, hemorrhagic stroke (symptomatic intraparenchymal, intraventricular, or subarachnoid hemorrhage), and stroke of undetermined type (ischemic or hemorrhagic) |
| Kennedy et al. 2007 | The secondary outcome: the combination of any stroke, myocardial infarction, and vascular death |
| Kim et al. 2019 | Composite of all stroke (ischemic and hemorrhagic), myocardial infarction, and vascular death |
| Wang et al. 2013 | Ischemic stroke, hemorrhagic stroke, myocardial infarction or death from cardiovascular causes, TIA |
| Wang et al. 2019 | Composite clinical vascular events: ischaemic/haemorrhagic stroke, transient ischaemic attack, myocardial infarction, or vascular death |
| Wang et al. 2021 | Composite vascular events: ischemic stroke, hemorrhagic stroke, TIA, myocardial infarction, or death from vascular causes |

AMI: Acute myocardial infarction; CT: Computed Tomography; DWI: diffusion-weighted imaging; MI: Myocardial infarction; MRI: Magnetic resonance imaging; TIA: transient ischemic attack.
